# Shunting Inhibition Improves Synchronization in Heterogeneous Inhibitory Interneuronal Networks with Type 1 Excitability Whereas Hyperpolarizing Inhibition Is Better for Type 2 Excitability

**DOI:** 10.1523/ENEURO.0464-19.2020

**Published:** 2020-05-08

**Authors:** Ruben A. Tikidji-Hamburyan, Carmen C. Canavier

**Affiliations:** Department of Cell Biology and Anatomy, Louisiana State University Health Sciences Center, New Orleans, LA 70112

**Keywords:** oscillations, phase amplitude coupling, phase locking, synchrony

## Abstract

All-to-all homogeneous networks of inhibitory neurons synchronize completely under the right conditions; however, many modeling studies have shown that biological levels of heterogeneity disrupt synchrony. Our fundamental scientific question is “how can neurons maintain partial synchrony in the presence of heterogeneity and noise?” A particular subset of strongly interconnected interneurons, the PV+ fast-spiking (FS) basket neurons, are strongly implicated in γ oscillations and in phase locking of nested γ oscillations to theta. Their excitability type apparently varies between brain regions: in CA1 and the dentate gyrus they have type 1 excitability, meaning that they can fire arbitrarily slowly, whereas in the striatum and cortex they have type 2 excitability, meaning that there is a frequency thresh old below which they cannot sustain repetitive firing. We constrained the models to study the effect of excitability type (more precisely bifurcation type) in isolation from all other factors. We use sparsely connected, heterogeneous, noisy networks with synaptic delays to show that synchronization properties, namely the resistance to suppression and the strength of theta phase to γ amplitude coupling, are strongly dependent on the pairing of excitability type with the type of inhibition. Shunting inhibition performs better for type 1 and hyperpolarizing inhibition for type 2. γ Oscillations and their nesting within theta oscillations are thought to subserve cognitive functions like memory encoding and recall; therefore, it is important to understand the contribution of intrinsic properties to these rhythms.

## Significance Statement

The collective, synchronized activity of neurons produces brain rhythms. These rhythms are thought to subserve cognitive functions such as attention and memory encoding and retrieval. We focus on fast-spiking (FS) basket cells, a subset of inhibitory interneurons. These neurons play an important role in brain rhythms. In some brain regions these neurons can fire arbitrarily slowly (type 1 dynamics) whereas in others they cannot fire below a minimum cutoff frequency (type 2 dynamics). We show that excitability type determines whether shunting or hyperpolarizing inhibition more effectively synchronizes the fast-oscillatory activity of networks of these neurons in the presence of heterogeneity and noise, and more effectively drives modulation of fast activity by slower oscillations.

## Introduction

γ Oscillations in cortex can arise from an interplay between excitatory pyramidal cells and inhibitory interneurons (Tiesinga and Sejnowski, 2009; Dumont and Gutkin, 2019) called pyramidal interneuronal network γ (PING). However, ING (with only inhibitory interneurons participating in the rhythm) can exist independently of PING. For example, during a selective visual attention task, phase locking of spikes in putative fast-spiking (FS) interneurons in primate area V4 to γ band oscillations in the local field potential (LFP) was strong both during the pre-stimulus period and during visual stimulation. In contrast, γ phase locking of putative pyramidal cells was strong only during visual stimulation that requires attention (Vinck et al., 2013). Similarly, in rodent areas S1 and V1, γ phase locking of putative FS cells is pervasive, whereas the putative E-cells are only recruited into γ during periods of locomotion and arousal (Vinck et al., 2015a,b; Perrenoud et al., 2016). Moreover, noncortical regions like the striatum, globus pallidus and substantia nigra pars reticulata are networks of inhibitory neurons with no pyramidal cells. Striatal FS interneurons have been implicated in γ rhythms (Berke, 2009; Sciamanna and Wilson, 2011). Here, we focus on ING with interneurons in the mean-driven, oscillatory regime, rather than the classical state of balanced excitation and inhibition. The stochastic population oscillator model posits that oscillations arise from the interactions between individual neurons in the balanced regime, such that the time between threshold crossings is exponentially distributed like the output of a Poisson process. However, that fluctuation-driven model is not compatible with the high-frequency firing rates exhibited by FS interneurons during γ in vivo (Bragin et al., 1995; Penttonen et al., 1998; Csicsvari et al., 1999), as noted in a previous review (Bartos et al., 2007).

PV+ FS basket interneurons are implicated in γ rhythms (Cobb et al., 1995; Bartos et al., 2007; Sohal et al., 2009; Gulyás et al., 2010; Varga et al., 2012), which in the hippocampus are thought to organize information for memory encoding and retrieval (Colgin et al., 2009; Bieri et al., 2014; Lasztóczi and Klausberger, 2014). γ Frequency (30–80 Hz) oscillations are thought to serve as substrates for working memory, conceptual categorization, and attention (Engel and Singer, 2001), and are altered in psychiatric disorders, for example, schizophrenia, dementia, and autism (Uhlhaas and Singer, 2006). PV+ FS basket cells play an important role in theta-nested γ (Wulff et al., 2009). Moreover, nesting of γ within theta has been proposed a substrate for episodic memory (Lega et al., 2016) and disruption of theta-nested γ has been proposed to explain deficits in spatial memory in temporal lobe epilepsy (Lopez-Pigozzi et al., 2016; Shuman et al., 2017).

Fast oscillations based on reciprocal inhibition have been repeatedly characterized as not being robust to heterogeneities in the network (Wang and Buzsáki, 1996; White et al., 1998; Bartos et al., 2007; Mann and Paulsen, 2007). Inhibitory neural networks generally lose synchrony as heterogeneity is increased in one of two ways, phase dispersion or suppression, depending on the ratio of the time constant for decay of inhibition to the population frequency (Chow et al., 1998; White et al., 1998). Here, we suggest that in networks with fast GABA_A_ synapses, which tend to favor the suppression regime over phase dispersion, cycle skipping is a way for a network to robustly preserve synchrony of individual spikes with the population by suppressing spikes that would have occurred too late to be in synchrony with the population. FS interneurons neurons were initially characterized as consistently having type 1 excitability in hippocampal area CA1 (Zhang and McBain, 1995; Wang and Buzsáki, 1996; Ferguson et al., 2013) and the dentate gyrus (Hu et al., 2010). Neurons with Hodgkin’s type 1 excitability are able to spike arbitrarily slowly, whereas those with type 2 excitability have an abrupt onset of repetitive firing that cannot be maintained below a threshold frequency (Canavier et al. 2006; Hodgkin, 1948; Izhikevich, 2007). More recently, FS neurons have been shown to consistently exhibit type 2 excitability in the medial entorhinal cortex (Tikidji-Hamburyan et al., 2015), striatum (Sciamanna and Wilson, 2011) and neocortex (Tateno et al., 2004). We systematically examine how the pairing of each excitability type with either hyperpolarizing or shunting inhibition affects cycle skipping synchronization in the presence of heterogeneity and noise, as well as synaptic delays. Simple 2D model neurons of each type were calibrated to have a very similar frequency current (F/I) curve, input resistance, time constant and action potential shape to isolate the consequences of excitability type alone on the robustness of synchronization in inhibitory interneuronal networks.

## Materials and Methods

### Single neuron models

The activation of the voltage-dependent sodium current was assumed to be fast and set to its steady-state value with respect to the membrane potential [$m_{\infty }(v)$]. The inactivation variable for the voltage-dependent Na^+^ current (h) was yoked to the variable (n) for the activation of the delayed rectifier K^+^ current, under the assumption that the slow time scale for these variables was similar.

(1) $$\begin{array}{l} C\frac{dv}{dt}=I-g_{L}\left(E_{L}-v\right)-g_{Na}m_{\infty }^{3}(v)\left(a+bn\right)\left(E_{Na}-v\right)\\ -g_{K}n^{4}\left(E_{K}-v\right)\\ \frac{dn}{dt}=\frac{n_{\infty }(v)-n}{\tau_{n}(v)} \end{array},$$

where: a=0.906483183915 and b=−1.10692947808 are the offset and slope for the h−n linear regression; $C=1\mu F/cm^{2}$ is a membrane capacitance; I is applied current in $\mu A/cm^{2}$; $g_{Na}=120$, $g_{K}=36$, and $g_{L}$ are conductances in $mS/cm^{2}$ for sodium, potassium, and leak currents with corresponding reversal potentials $E_{Na}=50mV$, $E_{K}=-77mV$, and $E_{L}$. $m_{\infty }(v)$ and $n_{\infty }(v)$ are steady state activations for sodium and potassium channels; and $\tau_{n}(v)$ is the time constant for potassium channel activation in ms:

(2) $$\begin{array}{l} m_{\infty }(v)=\frac{1}{1+\exp\left(-\frac{v+40}{9.5}\right)}\\ n_{\infty }(v)=n_{0}+\frac{1-n_{0}}{1+\exp\left(-\frac{v-v_{1/2}}{\theta }\right)}\\ \tau_{n}(v)=\tau_{0}+s_{\tau }\exp\left(-\frac{{\left(v-v_{0}\right)}^{2}}{\eta^{2}}\right) \end{array}.$$

**Figure 1.**
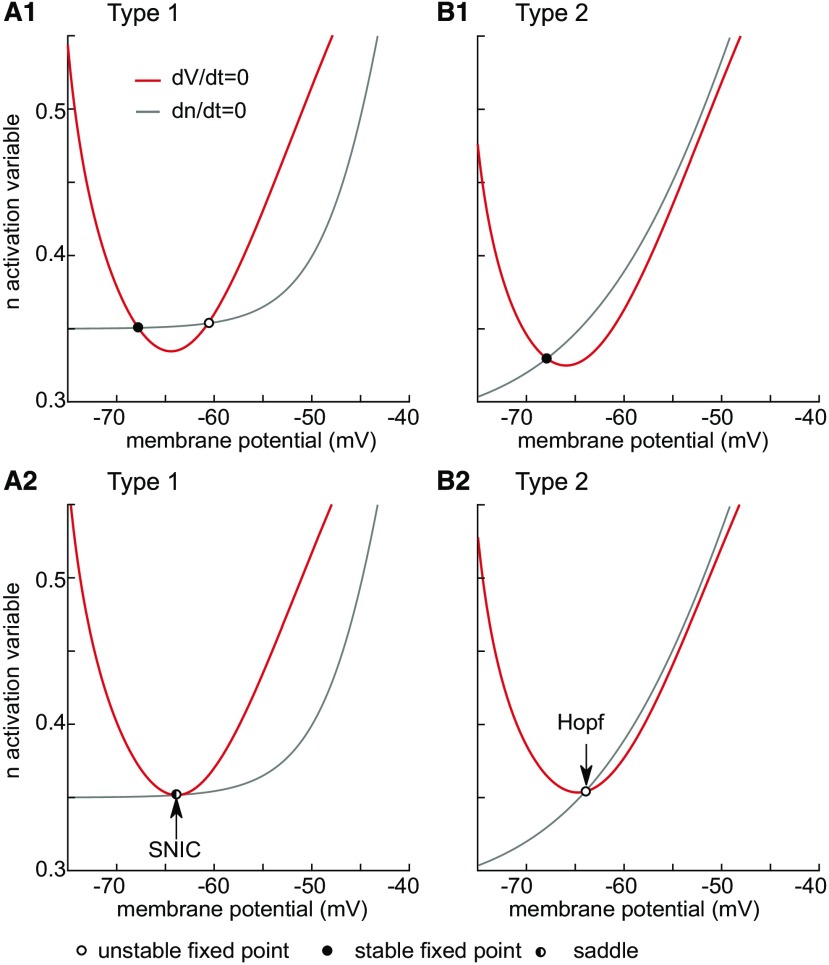
Phase plane analysis of model bifurcation structure. Red curves indicate the leftmost two branches of the cubic V-nullcline on which the rate of change of membrane potential is zero. The gray curve is the n-nullcline on which the rate of change of the slow variable is zero. Filled and open cycles are stable and unstable fixed points. The half-filled circle is the saddle node. Under the assumption of fast/slow dynamics, fixed points on the left branch are stable and those on the other branch shown are unstable. ***A***, Type 1. ***A1***, Quiescent neuron with no bias current has a stable resting potential (filled circle) on the left branch at −68 mV. Only one of the two fixed points on the unstable branch appear in this view, chosen to emphasize the bifurcation. ***A2***, The n-nullcline is tangent to the V-nullcline as a stable and unstable fixed point collide to form a saddle node with $I=1.38\mu A/cm^{2}$. The bifurcation is only a SNIC if a limit cycle is born simultaneously and emanates from the saddle node. ***B***, Type 2. ***B1***, Quiescent neuron with no bias current has a stable resting potential (filled circle) on the left branch at −68 mV. There is only a single fixed point. ***B2***, The Hopf bifurcation occurs as the fixed point loses stability as it moves onto the unstable branch at $I=2.11\mu A/cm^{2}$.

**Figure 2.**
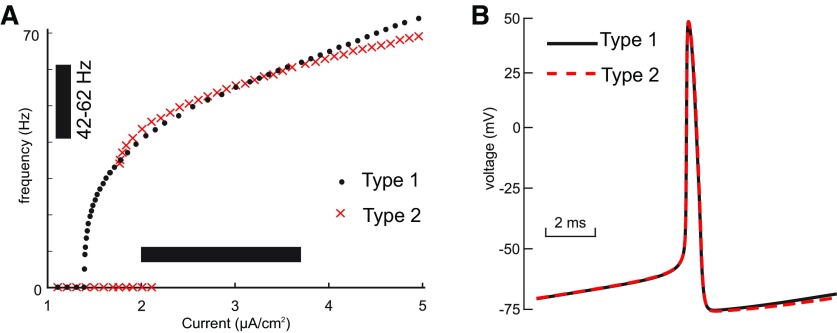
Model calibration. The parameters of the model for both types (parameters given in Table 1, attributes in Table 2) were adjusted to make the comparison as fair as possible. ***A***, Frequency/current (F/I) curves for type 1 (black dots) and type 2 (red crosses) overlap for the range of bias currents (horizontal bar) used in our heterogeneous networks. The vertical bar shows the range of frequencies exhibited at the heterogeneous bias current levels. The F/I curves were measured using current steps of sufficient duration to allow any transients to die out and establish a steady frequency. Arbitrarily slow frequencies can be obtained for type 1 (data not shown) near the bifurcation, but type 2 has a minimum cutoff frequency below which it cannot fire. Current steps were additive (the membrane potential was not returned to rest between steps). After stepping up, the current was stepped down to reveal the bistable region. The bistable range for type 2 is evident from the current values at which a zero-frequency quiescent solution coexists with a repetitively firing solution. This region was determined using XPPAUTO. ***B***, The spike shapes and the ISI are very similar for type 1 (black curve) and type 2 (red dashed curve); $I=2.85\mu A/cm^{2}$.

Model parameters $g_{L}$, $E_{L}$, $n_{0}$, $v_{1/2}$, θ, $\tau_{0}$, $s_{\tau }$, $v_{0}$, and η are different for type 1 and type 2 regimes and are given in Table 1 in order to achieve the bifurcations described in Fig. 1. These parameters were adjusted for the two excitability types to keep the input resistance ($\approx 2k\Omega \;cm^{2}$), time constant (≈2 ms), F/I curve (Fig. 2*A*), and spike shape (Fig. 2*B* and Table 2) as similar as possible. The shape of the steady state activation curve for the delayed rectifier is non-physiological because it does not go to zero (as in Franci et al., 2013). This phenomenological compromise was necessary to keep the input resistance of quiescent model neurons comparable and does not affect the dynamics of interest near the bifurcations.

**Table 1.** Parameters for type 1 and type 2 models

Type	$g_{L}(mS/cm^{2})$	$E_{L}(mV)$	$n_{0}$	$v_{1/2}(mV)$	θ(mV)	$\tau_{0}(ms)$	$s_{\tau }(ms)$	$v_{0}(mV)$	η(mV)
1	0.3	–54.3	0.35	–40.0	4.0	0.46	3.5	–60.5	35.9
2	0.1	–39.0	0.28	–44.5	9.0	0.5	5.0	–60.0	30.0

**Table 2:** Characteristics of membrane passive properties and spike shape for type 1 and type 2 models

	Type 1	Type 2
Resting potential (mV)	–67.78	–67.91
Positive input resistance (Ω/cm^2^)	1741	2032
Negative input resistance (Ω/cm^2^)	1761	2027
Spike threshold (mV)	–44.34	–44.81
Spike width on half height (ms)	0.39	0.40
Spike height (mV)	88.00	88.39
After hyperpolarization (mV)	31.01	30.54

### Detailed theoretical dynamical description of bifurcations

The voltage nullcline for excitable neurons is N-shaped with three branches, but to emphasize the bifurcation point where repetitive spiking is born, only the stable left and unstable middle branch and their associated fixed points are shown in Figure 1. For type 1 at rest (Fig. 1*A1*), in addition to the stable fixed point that determines the resting potential, there is an unstable fixed (open circle) point on the middle branch, as well as another one on the unstable branch that is not shown. As the applied current is increased to $I\approx 1.38\mu A/cm^{2}$ the stable and unstable fixed points shown collide (Fig. 1*A2*) and form a saddle node (circle half filled). The neuron is left with no stable resting potential, but a closed curve representing repetitive spiking (a limit cycle; data not shown) coalesces around the rightmost unstable fixed point on the unstable branch (also data not shown) and intersects with the saddle node. The technical term for the onset of spiking is a saddle-node-on-an-invariant-limit-cycle (SNIC) bifurcation (Izhikevich, 2007; Ermentrout and Terman, 2010). The intersection of the limit cycle with the saddle node gives rise to a trajectory with infinite period. As the applied current is increased any further, the trajectory must pass through a gap between the two nullclines; the rate of passage though this bottleneck is arbitrarily slow with increasing proximity to the bifurcation, hence the arbitrarily slow frequencies obtainable by type 1 neurons shown in Figure 2*A*, black dots. The intuitive explanation for slow trajectories is that the rate of change for both *v* and n is zero on the nullclines, and since the gap is very close to both nullclines, the rate of change for both variables is very slow.

In contrast, for type 2 excitability, the Hopf bifurcation occurs when the lone stable fixed point corresponding to the rest potential loses stability (Fig. 1*B1*). When the applied depolarizing current reaches $I\approx 2.11\mu A/cm^{2}$, the fixed point (Fig. 1*B2*, open circle) moves past the trough of the nullcline onto the unstable branch of the voltage nullcline. The Hopf bifurcation is subcritical (Guckenheimer and Holmes, 1997; Izhikevich, 2007; Ermentrout and Terman, 2010) because in the range of input currents between $I\in \left[1.74,\;\;2.11\right]\mu A/cm^{2}$ the model exhibits bistability between stable limit cycle corresponding to repetitive spiking and a stable fixed point (Fig. 2*A*, overlap in the red cross at 0 frequency and at a nonzero frequency). These attractors are separated by unstable limit cycle (data not shown). The stable and unstable limit cycles collide and annihilate each other in a saddle node of periodics at $I\approx 1.74\mu A/cm^{2}$. Excitability type is defined as the response of a quiescent neuron as the applied current is increased (Hodgkin, 1948); the sudden onset of spiking as the applied current is increased occurs at ∼30 Hz (x’s in Fig. 2*A*) when the quiescent state loses stability as shown in Figure 1*B*.

### Network

For all simulations we used 300 neurons of the same excitability type, connected by bi-exponential inhibitory synapses. In the network, the input current for each neuron is given by the following equations:

(3) $$\begin{array}{l} I_{i}=I_{0,i}+I_{n,i}+\left(b_{i}-a_{i}\right)\left(v_{i}-E_{syn}\right)+g_{mod}(t)\left(v_{i}-E_{syn}\right)\\ \frac{da_{i}}{dt}=\kappa \sum_{j}g_{ij}\delta \left(t-t_{j}-\delta_{ij}\right)-\frac{a_{i}}{\tau_{1}}\\ \frac{db_{i}}{dt}=\kappa \sum_{j}g_{ij}\delta \left(t-t_{j}-\delta_{ij}\right)-\frac{b_{i}}{\tau_{2}} \end{array},$$

where $v_{i}$ and $I_{i}$ are membrane potential and input current of $i^{th}$ neuron; $I_{0,i}$ is an applied current; $I_{n,i}=\sigma N(0,1)$is a noise current with an independent random process with zero mean and unit variance for each neuron N(0,1). These processes were sampled every 0.1 ms, and the current was linearly interpolated between these times to produce consistent results regardless of the time step.

Fast, ionotropic inhibition in the central nervous system in generally mediated by GABA_A_ receptors, with chloride ions as the charge carrier. The reversal potential of these channels depends on the intracellular concentration of chloride. In quiescent neurons, if the synaptic reversal potential is negative to the resting potential such that a prominent hyperpolarizing synaptic potential can be observed, then the inhibition is hyperpolarizing. On the other hand, if the reversal potential is negative to the spike threshold but is close to the resting potential such that the main effect is a change in conductance and a prominent hyperpolarizing synaptic potential is not observed, the inhibition is shunting. For oscillatory neurons, the membrane potential during the interspike interval (ISI) substitutes for the resting potential (Mann and Paulsen, 2007). Shunting inhibition is sometimes defined as an increase in synaptic conductance in the absence of an obvious change in membrane potential (Paulus and Rothwell, 2016). In our model neurons, a synaptic reversal potential of −75 mV produces hyperpolarization whereas −65 mV does not, so synaptic reversal potential $E_{syn}$was set to −75 mV for hyperpolarizing and –65 mV for shunting inhibition. The synaptic rise and fall time constants were $\tau_{1}=1\;ms$and $\tau_{2}=3\;ms$, respectively. The conductance ($g_{ij}$) and conduction delay ($\delta_{ij}$) between the $i^{th}$ and $j^{th}$ neurons are given in units of mS/cm^2^ and ms, respectively; δ()is Dirac’s δ function; and κ is a normalization constant to keep peak of $b_{i}-a_{i}$ equal to $g_{ij}$.

For both steady-state and sinusoidally modulated network oscillations, $I_{0,i}$ were drawn from uniform distribution with the range $\left[2,\;\;3.8\right]\mu A/cm^{2}$ providing a distribution of intrinsic frequencies with a 20-Hz range (Fig. 2*A*, vertical and horizontal bars). In steady-state oscillations regime inhibitory modulatory conductance $g_{mod}$was set to zero. In a contrast, for modulated network oscillations $g_{mod}$ was sinusoidally modulated: $g_{mod}(t)=\frac{{\bar{g}}_{mod}}{2}\left[1-\cos\frac{2\pi }{P}t\right]$, where P is the period, and ${\bar{g}}_{mod}$is the amplitude of modulation.

Connections in the network were sparse and random with probability p=0.133 of connection between any given the $i^{th}$ and $j^{th}$ neurons. For all simulations presented here, conduction delays were uniformly randomly distributed between 0.7 and 3.5 ms.

### Numerical simulations and bifurcation analysis

The bifurcation analysis was performed in XPPAUTO (Ermentrout, 2002). The network models were implemented as Python 2.7 script for the simulation package NEURON (Hines and Carnevale, 1997). The code/software described in the paper is freely available online at https://senselab.med.yale.edu/ModelDB/showmodel.cshtml?model=259366#tabs-1]. The integration time step was constant at 0.01 ms. Synaptic activation initially was set to zero for all simulations. In order to better explore the potential dynamics states of the networks, the instantaneous value of the membrane potential of each neuron was initialized randomly from a normally distribution with mean –50 mV and SD of 20 mV. The slow n variable was initialized at the steady-state value for the membrane potential. The data presented on vector strength, participation, coefficient of variation (CV) of participation and total suppression for networks biased in the oscillatory regime were averaged over ten trials at each parameter setting for runs of duration 2.5 s with the initial 500 ms ignored to minimize the effects of transients. Each trial had its own random connectivity pattern, random initialization of the state variables, random distribution of bias currents, random delay distribution, and random noise sources. For the sinusoidal drive simulations, the phase amplitude coupling was averaged over 20 periods of sinusoidal drive, again averaged over 10 trials as described above, but no transients were deleted. The phase of each spike within a cycle used was calculated using the length of that particular cycle. Cycle lengths are variable and were computed using the peaks in the population rate as described in Tikidji-Hamburyan et al. (2015). The phase was used to construct the vectors for the vector strength calculation.

In some figures we applied an additional source of variability. We applied a multiplicative scale factor ($F_{i}$) to the rates of change of both variables. Since the Gaussian noise term simulates Brownian motion in the membrane potential, in which distance is proportional to the square root of time, the noise term is then divided by the square root of the scale factor. Taken together, these manipulations simply scale the intrinsic frequency since all intrinsic (but not synaptic) processes are sped up or slowed down equally.

### Measure of phase amplitude coupling

Since we apply the theta drive, there is no uncertainty with respect to the phase of the theta oscillation, in contrast to the uncertainty in experimental data such as LFPs or the EEG. This simplified our analysis. Moreover, since we apply the exact same amplitude of theta modulation to different networks, we were interested not only in the tightness of the coupling of the theta drive and the evoked nested γ oscillation, but also in the magnitude of the γ oscillations. Therefore to quantify the coupling between theta phase and γ amplitude, we choose the mean vector length (MVL; Canolty et al., 2006; Hülsemann et al., 2019), but without normalization of the amplitude. The vectors consisted of the known theta phase with the magnitude given by the amplitude of the γ envelope determined using the Hilbert transform of the simulated LFP. The simulated LFP was the synaptic inhibitory current summed over the network. The sum of these vectors produces a vector strength that is not bounded between 0 and 1, but which does accurately reflect the amplitude of the nested γ oscillations evoked by a constant sinusoidal stimulus at theta frequency, and the preferred theta phase. The normalized vector strength shows only how strongly the γ envelope is locked to the preferred theta phase. The unnormalized version takes into account the actual amplitude of the γ envelope.

## Results

### Phenomenological model of type 1 and type 2 excitability

Electrophysiologists frequently characterize neurons using the steady state current/voltage (IV) curve and the F/I curve. These measures are useful to quantify the excitability of a cell. However, the phase-plane portrait technique (Edelstein-Keshet, 2005; Strogatz, 2015) gives a more precise description of the basis for excitability type, which we link here to the underlying bifurcation structure (see Discussion, Generality and limitations). In a 2D system, the rate of change of each variable defines a vector at each point in the plane, called a vector field; a knowledge of the vector field allows the prediction of the trajectory in the plane. We used a 2D reduction (Rinzel, 1985) of the Hodgkin–Huxley model (Hodgkin and Huxley, 1952), because this 2D system with one fast variable (membrane potential, v) and one slow variable (n) is amenable to phase plane analysis under fast/slow assumptions (Bertram and Rubin, 2017). Fast/slow assumptions means that there is time scale separation and the fast variable, membrane potential, changes much more rapidly than the slow variable, with clear implications for movement in the phase plane.

A steady state IV curve that intersects the zero current axis in a single point is often associated with type 2 excitability, whereas one with multiple intersections is often associated with type 1; the same is true for the intersections of the nullclines in the phase-plane portrait (Rinzel and Ermentrout, 1998). However, as stated in Discussion, Generality and limitations, there are exceptions. Figure 1 shows a phase-plane analysis of type 1 (Fig. 1*A*) and type 2 (Fig. 1*B*) excitability. In the phase plane, fixed points occur at the intersection of the n-nullcline (gray curve), which is simply the steady state activation curve for the n activation variable, and the voltage nullcline at rest (red curve), which is the set of values of n and v for which the net ionic current plus any applied current is zero. For this fast/slow system the leftmost branch is stable, and the middle branch is unstable due to the regenerative, autocatalytic sodium current. At rest (with no applied current), the fixed point (filled circle) on the left branch is stable and determines the resting potential at −68 mV for both the type 1 (Fig. 1*A1*) and type 2 (Fig. 1*B1*) cases. An important difference is that for type 2, there is a single fixed point at all values of applied current, whereas for type 1 the number of fixed points depends on the level of the applied current.

### Steady state synchrony in oscillatory networks

We constructed networks of 300 sparsely and randomly connected neurons with heterogeneity in frequency by distributing the bias current uniformly along a region of the F/I curve that spanned a 20-Hz range (Fig. 2*A*, black bars). The conduction delays between neurons were also uniformly distributed between 0.7 and 3.5 ms. In a homogeneous network with strong but fast inhibitory synapses, delays on the short end of this range favor a solution with two subclusters in antiphase, whereas delays at the longer end of the range favor global synchrony of a single cluster (Tikidji-Hamburyan et al., 2019). Using a mixture of delays results in solutions that are not obviously one or two clusters, but are transitional between these two extremes.

### Steady state synchrony in oscillatory networks, hyperpolarizing inhibition

**Figure 3.**
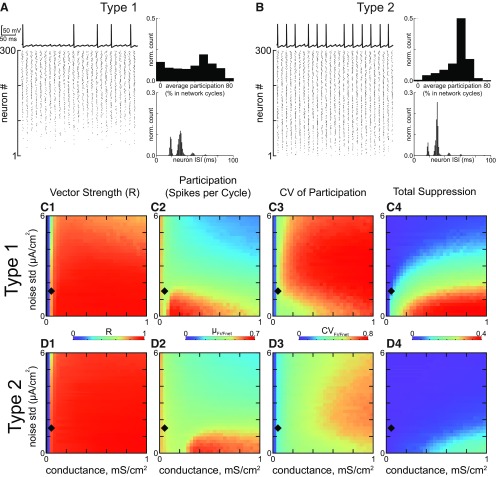
Steady state synchronization of heterogeneous inhibitory interneuronal networks with hyperpolarizing inhibition. ***A***, Networks of type 1 neurons with synaptic conductance 0.05 mS/cm^2^ and noise SD 1.5 µA/cm^2^, indicated by black diamonds in ***C***, ***D***. Bottom left, Raster plot for 300 neurons, ordered by the level of applied depolarizing bias current, with the most depolarized neurons at the top. Top left, Representative membrane potential trace for an individual neuron that clearly exhibits a subthreshold oscillation due to network activity and cycle skipping. Bottom right, Histogram of ISIs across the population. Top right, Histogram of average participation for all neurons in the network. ***B***, Networks of type 2 neurons with the same parameters as in ***A***. The four panels are the same as in ***A***. ***C***, 2D parameter sweep in conductance strength and noise SD for type 1 networks. Heatmaps from left to right, vector strength R of synchronization of individual spikes with the network oscillation (***C1***); average participation for neurons that are not totally suppressed calculated as the mean µ_Fr/Fnet_ of the frequency of spiking neurons normalized by the population frequency (***C2***); the CV of the participation CV_Fr/Fnet_ (***C3***); and the fraction of completely suppressed neurons (***C4***). ***D***, 2D parameter sweep for type 2 networks. Heatmaps are the same as in ***C***.

Figure 3*A*,*B*, top left, shows representative traces from network simulations with hyperpolarizing inhibition showing how individual neurons of both types in noisy, sparsely connected and heterogeneous networks skip random cycles. However, neurons in both populations remain synchronized with the population oscillation when they do fire, which is evident from raster plots Figure 3*A*,*B*, bottom-left. The cycle skipping is evident in the histograms of ISIs (Fig. 3*A*,*B*, bottom right) across all neurons. There are peaks at the network period and at integer multiples of the cycle period, corresponding to how many cycles were skipped during that interval. Neurons in the raster plots are sorted by bias current with the fastest firing cells at the top. This example shows that the slower firing neurons at the bottom of the raster plots have a much greater likelihood of being completely or partially suppressed for type 1 networks compared with type 2 networks. The histogram of the average number of network cycles each individual neuron participated (Fig. 3*A*,*B*, top right) in is much flatter for type 1 than type 2, because the type 2 histogram is skewed to higher participation rates.

This tendency for greater suppression of type 1 neurons with hyperpolarizing inhibition was preserved across a large range of the 2D parameter space of synaptic conductance strength and SD of the additive Gaussian current noise, as shown by the heatmaps in Figure 3*C*,*D*. The diamond in the heatmap indicates the parameters used to generate the raster in Figure 3*A*,*B*. The leftmost heatmap gives the vector strength measure of population synchrony. For both type 1 (Fig. 3*C1*) and type 2 (Fig. 3*D1*), a minimum amount of conductance is required for synchrony (blue strip at the left is unsynchronized). Both networks perform fairly well for synchrony of spikes with the population rhythm over this range, although type 2 performs a little better at high noise levels and stronger conductance. The vector strength measure of synchrony is quite high, and exceeds 0.8 almost everywhere. The average spikes per cycle (excluding neurons that are completely suppressed) decreases with increasing noise, but again similar for the two types of networks (Fig. 3*C2*,*D2*). The major difference is evident in the heatmaps (Fig. 3*C3*,*D3*) for the CV for participation across the population. As expected from the participation histograms shown in Fig. 3*A*,*B*, top right of each, the CV is greater for type 1, reflecting the greater abundance of partially suppressed neurons. The CV is in the range 0.5–0.8 for type 1 compared to 0.3–0.6 for type 2. This discrepancy would be even greater had we included the factions of completely suppressed neurons illustrated in the rightmost histograms (Fig. 3*C4*,*D4*), which clearly show a far greater fraction of completely suppressed neurons for type 1. Stronger noise desynchronizes and stronger inhibition promotes suppression, so outside the regime of interest neither type performs well, and the difference between type 1 and 2 fades.

**Figure 4.**
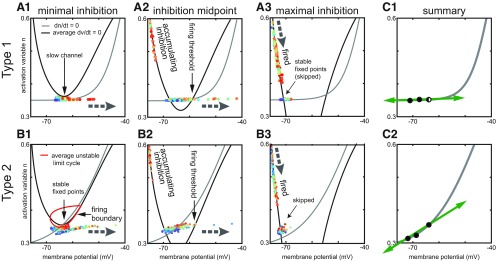
Phase plane analysis of heterogeneous inhibitory interneuronal networks with hyperpolarizing inhibition. Large gray dashed arrows show the local direction of motion in the vector field. Other thin black arrows simply point out a specific feature. These phase plane portraits are similar to those in Figure 1 with two exceptions. First, the V-nullcline (black curves) shown is an average across the heterogeneous population. Thus, the fixed points shown are also an average, and actually differ for each neuron. Second, the time-varying levels of inhibition between the neurons causes the position of the average V-nullcline to fluctuate in time. The slow n-nullcline (gray) is constant for each type. Each dot represents the current position of one neuron in the network, with the fastest neurons in red and the slowest in blue (there is also a contribution to the dynamics by the number of active inhibitory inputs to each neuron, which cannot be inferred from the color code). Movement in the horizontal direction is fast compared with that in the vertical direction because the slow variable is plotted along the y-axis. ***A***, Type 1. ***A1***, Minimal inhibition creates a slow channel between the nullclines (vertical arrow). ***A2***, Inhibition midpoint. As neurons escape to the right (horizontal arrow shows direction of motion) and fire an action potential, inhibition accumulates and moves the average V-nullcline down. The right branch of the V-nullcline is the firing threshold (vertical arrow). ***A3***, Maximal inhibition. The population is segregated into two groups. One group that fired on the most recent network cycle slowly moves down (thick arrow at top shows direction of motion) along the stable left branch of the average V-nullcline. The other group (diagonal arrow) skipped this cycle and tends to line up along the flat portion of the n-nullcline. ***B***, Type 2. ***B1***, Minimal inhibition. In contrast to the absence of a fixed point in ***A1***, there is a stable fixed point (technically fixed points, vertical arrow) at the intersection of the average V-nullcline and the n-nullcline. This fixed point is surrounded by an unstable limit cycle (red curve) that forms the boundary between spiking and quiescent trajectories. ***B2***, Inhibition midpoint. As in ***A2***, inhibition accumulates as neurons escape to the right and fire action potentials. The right branch of the V-nullcline is now the firing threshold (vertical arrow). ***B3***, Inhibition maximum. As in ***A3***, two groups are evident corresponding to those that fired and those that skipped. ***C***, Summary. ***C1***, This panel shows that the movement of the fixed point as inhibition waxes and wanes is (leftward) in the fast, horizontal direction for type 1. ***C2***, In contrast, the downward and leftward movement of the fixed point for type 2 has a component in the slow direction, which helps equalize the opportunity of slow neurons to fire as compared with fast neurons.

The 2D phase plane representation of network activity shown in Figure 4 illustrates the mechanism underlying greater suppression of type 1 compared with type 2 neurons in networks with hyperpolarizing inhibition. The n-nullcline for the slow variable (gray curve) was constant in time and across the population. On the other hand, the membrane potential V-nullcline in the absence of inhibition was different for every neuron because of the heterogeneity in applied bias current. This required averaging the V-nullcline across the population. Moreover, the level of inhibition is a third state variable per neuron, and the average level of inhibition in the network varies in time. Therefore, the 2D phase plane representation of the V-nullcline (black curve) actually constitutes a movie (Movie 1 for type 1 and Movie 2 for type 2). The movies provide the clearest picture of the dynamics, but snapshots of the time-varying portrait are given for minimal, half-amplitude, and maximal inhibition in Figure 4*A1–A3*,*B1–B3* for types 1 and 2, respectively. Each dot represents the current position of a neuron in this phase space, with the neurons with the highest depolarizing bias current shown in red and those with the least in blue.

Movie 1. Dynamics of the type 1 population with hyperpolarizing inhibition. Left plots from top to bottom, Population raster plot: color code indicates amplitude of excitatory drive. Population voltage traces: population slow variable traces; averaged synaptic conductance of the population. Right, Phase plane analysis. Position of each neuron in the state space is marked by a color cycle. Color code is the same as in top left plot. Blue solid line indicates average instantaneous voltage nullcline. Dashed blue line indicates voltage nullcline in the absence of inhibition. Red dashed line shows slow variable nullcline. 10.1523/ENEURO.0464-19.2020.video.1

Movie 2. Dynamics of the type 2 population with hyperpolarizing inhibition. Plots and lines are the same as for Movie 1. An additional black dash-dot curve indicates unstable limit cycle which breaks and turns in quasi-threshold. 10.1523/ENEURO.0464-19.2020.video.2

For type 1, at minimum (but nonzero) inhibition, most neurons are in a near threshold regime (Fig. 4*A1*,*B1*). The average nullcline portrait is close to the SNIC bifurcation at which the two indicated fixed points collide and destroy each other. For type 1 networks: a slow channel (arrow) between the two nullclines arises as the system approaches the SNIC bifurcation (Fig. 1*A2*). By definition, the rate of change of a variable is zero on its nullcline, therefore close to the nullcline, the rate of change is quite slow. In the channel, the rate of change of both variables is quite slow, and the level of bias current imposes order on the trajectories, with the fastest cells positioned nearest the firing threshold. As neurons escape from the channel and fire action potentials, inhibition accumulates as the IPSPs from the spiking neurons (shown moving down the left branch of the V-nullcline) summate in Figure 4*A2*. The accumulating inhibition moves the average V-nullcline down and to the left, closing the channel and creating a stable fixed point that enables a resting potential (as well as an unstable one; as in Fig. 1*A1*) at the intersection with the n-nullcline. The unstable branch of the V-nullcline (arrow indicating firing threshold points to this branch) forms a boundary that separates neurons into two groups. Neurons whose trajectory has already moved to the right of this branch will fire an action potential, but those that fall on the left side of this boundary when the channel closes will skip this network cycle. In general, the neurons that skip are the slower neurons; they will move leftward toward the stable fixed point. The two groups are clearly shown in Figure 4*A3* at the point of maximal inhibition after all spiking neurons have fired on a given cycle. The group labeled fired is recovering from the after-hyperpolarizing potential (AHP) along the stable V-nullcline branch. The group labeled skipped is trapped on their fixed point and did not participate in the previous cycle. The intersection of the average nullclines is the average fixed point; the actual fixed point for the slower neurons lies to the left of the average and for the faster neurons it lies to the right. The important point is that the neurons tend to line up from left to right with the faster neurons on the right. As the maximal inhibition decays, the phase portrait reverts to Figure 4*A1*; the faster on the right neurons have a clear advantage because they are more likely to escape the slow channel before it closes than the slower neurons. The slower neurons are much more likely to be suppressed as is evident in the raster plot in Figure 3, compare *A*, *B*, and the over greater suppression for type 1 compared with type 2 that is evident in the two rightmost heatmaps in Figure 3*C*.

The phase portrait for minimal inhibition for type 2 in Figure 4*B1* shows that most neurons are in a near threshold regime as in Figure 4*A1* for type 1. The average nullcline portrait is close to a bifurcation as in Figure 4*A1*, because the average fixed point requires only a small amount of additional excitation to move to the unstable branch of the V-nullcline as shown in Figure 1*B*. However, the vector flow near a subcritical Hopf bifurcation is completely different compared with the flow near a SNIC: there is no slow channel. Instead, as shown in Figure 2*A*, near a subcritical Hopf bifurcation, a bistable region exists in which quiescence at a stable fixed point (arrow) co-exists with repetitive spiking at the same value of net applied current. Whether the neuron is silent or active depends on recent history, meaning the current location of the trajectory. Specifically, the red closed curve in Figure 4*B1* is an unstable limit cycle that divides the neurons into two groups. Those inside the red curve will spiral into the stable fixed point at its center, whereas those outside will curve around it to fire an action potential. Thus, the mechanism for cycle skipping has a component that results from the intrinsic dynamics of the circular flow around a Hopf bifurcation; the circular flow in the phase plane results from oscillatory dynamics in the time domain due to the emergence of complex eigenvalues in the linearized solution evaluated at the fixed point. In contrast to the SNIC bifurcation in Figure 4*A*, there is no bias toward faster cells, all neurons are almost equally likely to fall outside of the quiescent zone and fire an action potential. Figure 4*B2* shows that as neurons move to the right and fire actions potentials, inhibition accumulates as in Figure 4*A2* and again moves the V-nullcline downward, shifting the fixed point down and to the left. The unstable limit cycle opens up into a quasi-threshold (data not shown) that then merges with the unstable branch of the V-nullcline, labeled firing threshold. As Figure 4*A3*,*B3* clearly shows two groups of neurons at the point of maximal inhibition, after all spiking neurons have fired on a given cycle. However, the group labeled skipped is still approaching their fixed points, and the distribution of these fixed points will be slanted rather than flat due to the sharper angle of the n-nullcline at the intersection. The sharper slope allows the n-nullcline to avoid the other branches of the V-nullcline enabling a Hopf bifurcation to underlie type 2 excitability, whereas multiple intersections are required for the SNIC with type 1 excitability.

Figure 4 *C1*,*C2* summarizes the movement of the stable fixed point due to changes in the level of inhibition. For type 1 in Figure 4*C1*, the requirement that the n-nullcline be tangent to the V-nullcline at the SNIC bifurcation imposes a relatively flat slope on the n-nullcline near the intersection, which causes hyperpolarizing inhibition to move the fixed points horizontally, but not vertically (green arrows). The horizontal direction is the fast direction of the dynamics, therefore the trajectories remain on or near the n-nullcline with constant order. The firing threshold splits the population into spiking neurons on the right and suppressed skipping neurons on the left. The fixed points of the neurons are ordered with those of the neurons with slower firing frequencies (lower bias current) on the left indicating more hyperpolarized fixed points and those corresponding to faster firing frequencies on the right, and the movement of the fixed points as inhibition waxes and wanes does not substantially alter their distribution. Therefore, neurons with the slower intrinsic firing frequencies have a strong tendency to be suppressed with hyperpolarizing inhibition. In contrast, Figure 4*C* shows the steeper slope of the n-nullcline causes hyperpolarizing inhibition to move all the fixed points downward as well as leftward (green arrow). The trajectories in Figure 4*B3* for the skipping neurons do not follow the fixed point as Figure 4*A3*, because downward movement is in the slow direction. This downward and leftward trend moves most trajectories out of the unstable limit cycle when it forms in Figure 4*B1* around the rightmost fixed point in Figure 4*C2*. This allows all neurons access to the fast-curved vector fields that push them to an action potential trajectory, the composition of the neurons that spike on any given cycle is more evenly distributed throughout the population. This phenomenon has a contribution form anodal break excitation (Fitzhugh, 1976), also called postinhibitory rebound (Perkel and Mulloney, 1974), and explains why suppression is less prominent in the raster plot in Figure 3, compare *B*, *A*, and the rightmost heatmaps of Figure 3, compare *D*, *C*.

### Steady state synchrony in oscillatory networks, shunting inhibition

**Figure 5.**
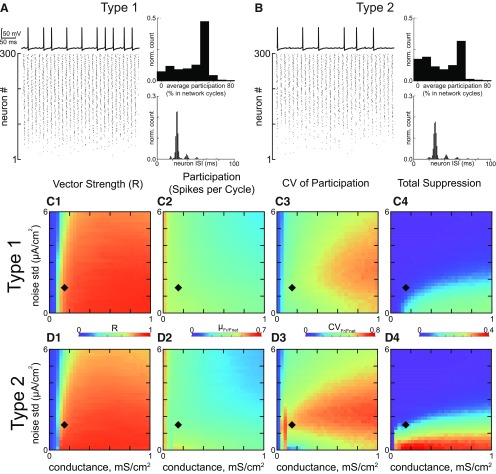
Steady state synchronization of heterogeneous inhibitory interneuronal networks with shunting inhibition. ***A***, Networks of type 1 neurons with synaptic conductance 1.5 mS/cm^2^ and noise SD 1.5 µA/cm^2^, indicated by black diamonds in ***C***, ***D***. Bottom left, Raster plot for 300 neurons, ordered by the level of applied depolarizing bias current, with the most depolarized neurons at the top. Top left, Representative membrane potential trace for an individual neuron that clearly exhibits a subthreshold oscillation due to network activity and cycle skipping. Bottom right, Histogram of ISIs across the population. Top right, Histogram of average participation for all neurons in the network. ***B***, Networks of type 2 neurons with the same parameters as in ***A***. The four panels are the same as in ***A***. ***C***, 2D parameter sweep in conductance strength and noise SD for type 1 networks. Heatmaps from left to right give (***C1***) the vector strength R of synchronization of individual spikes with the network oscillation; (***C2***) the average participation for neurons that are not totally suppressed calculated as the mean µ_Fr/Fnet_ of the frequency of spiking neurons normalized by the population frequency; (***C3***) the CV of the participation CV_Fr/Fnet_; and (***C4***) the fraction of completely suppressed neurons. ***D***, 2D parameter sweep for type 2 networks. Heatmaps are the same as in ***C***.

The results in the previous section were for hyperpolarizing inhibition. For both type 1 and type 2, the bifurcation that gives rise to spiking occurs at about −65 mV. Hyperpolarizing inhibition with a reversal potential of −75 mV hyperpolarizes the membrane at most points during the ISI except during the trough of the AHP. Shunting inhibition with a reversal potential of −65 mV does not produce big changes in the membrane potential. Figure 5 shows the same simulations as Figure 3 except for versus hyperpolarizing inhibition. Cycle skipping to preserve population synchrony is still prominent in both types, as evidenced by the single neuron traces in the top left of 5*A* and *B* and by the peaks at integer multiples of the network frequency in the ISI histograms at lower right. The leftmost heatmaps (Fig. 5*C1*,*D1*) confirm population synchrony is robust for both types, with generally lower participation (Fig. 5*C2*,*D2*) than for hyperpolarizing inhibition at the same parameter values. In general, neurons that are not completely suppressed fire on average every other cycle. However, type 2 networks clearly lose their superior resistance to suppression, as evidenced by the rasters in Figure 5*A* vs 5*B*. Moreover, the histogram of participation for individual neurons is much flatter in the top right of Figure 5*B* for type 2 with shunting inhibition compared with top right of Figure 3*B* for type 2 with hyperpolarizing inhibition, and in fact is slightly flatter than the histogram for type 1 with shunting inhibition in the top right of Figure 5*A*. The two rightmost heatmaps once again show that these results are general, with CVs of participation that are slightly larger for type 2 (Fig. 5*D3* vs *C3*), as well as more suppressed neurons at lower noise values for type 2 (Fig. 5*D4* vs *C4*).

**Figure 6.**
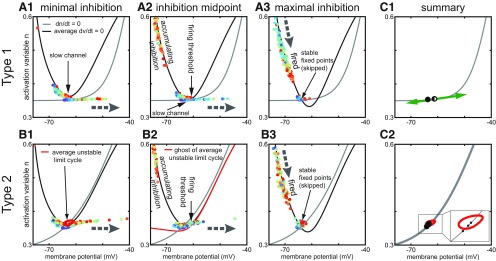
Phase plane analysis of heterogeneous inhibitory interneuronal networks with shunting inhibition. These phase plane portraits are similar to those in Figure 4 except the inhibition is now shunting instead of hyperpolarizing. Large gray dashed arrows again show the local direction of motion in the vector field. Other thin black arrows simply point out a specific feature. The average V-nullcline (black curves) and the slow n-nullcline (gray) are shown for each type. Each again dot represents the current position of one neuron in the network, with the fastest neurons in red and the slowest in blue. ***A***, Type 1. ***A1***, Minimal inhibition again creates a slow channel between the nullclines (vertical arrow). ***A2***, Inhibition midpoint. The right branch of the V-nullcline is the firing threshold (vertical arrow). As neurons escape to the right (horizontal arrow shows direction of motion) and fire an action potential, inhibition again accumulates and moves the average V-nullcline down. ***A3***, Maximal inhibition. The population is again segregated into two groups. However, the group that skipped this cycle (diagonal arrow) does not line up as clearly as in Figure 4*A* along the flat portion of the n-nullcline. ***B***, Type 2. ***B1***, Minimal inhibition. In contrast to the absence of a fixed point in ***A1***, there is a stable fixed point at the intersection of the average V-nullcline and the n-nullcline. This fixed point is again surrounded by an unstable limit cycle (red curve) that forms the boundary between spiking and quiescent trajectories. ***B2***, Inhibition midpoint. As in ***A2***, inhibition accumulates as neurons escape to the right and fire action potentials. However, in this case, the ghost of the unstable limit cycle unfurls into a quasi-threshold (red curve) and separates spiking and skipping trajectories (vertical arrow labeling the red curve as the firing threshold). ***B3***, Inhibition maximum. As in ***A3***, two groups are evident corresponding to those that spiked and those that skipped (diagonal arrow). ***C***, Summary. ***C1***, This panel shows that there is less leftward movement of the fixed point as inhibition waxes and wanes in the fast, horizontal direction for type 1 compared with in Figure 4*C1*. ***C2***, The fixed point for type 2 tends to trap trajectories inside the unstable limit cycle (red curve, see blowup in inset), which enforces skipping.

Figure 6 shows a phase plane analysis of the dynamics in a manner exactly analogous to Figure 4. Movies 3, 4 correspond to the phase portraits of type 1 and type 2 for shunting inhibition, respectively. For type 1, in Figure 6*A*, the V-nullcline does not move as much with a shunting inhibitory synaptic reversal potential of −65 mV as compared with a hyperpolarizing one of −75 mV. Therefore, the fixed point moves very little (Fig. 6*C1*), keeping neurons that skipped near the firing threshold. The distribution of the dots representing neural trajectory are more compressed along the n-nullcline in Figure 6*A3*, which reduces the advantage of the fastest neurons with the rightmost fixed points in escaping for the channel, hence the decrease in suppression. The V-nullclines and the corresponding fixed also move less with inhibition in Figure 6*B* for type 2. In contrast to Figure 4*B2*, the quasi-threshold described above (Fig. 6*B2*, red curve) is now visible as the “ghost” of the unstable limit cycle at the inhibition midpoint. Moreover, the inset in Figure 6*C2* reveals that the synaptic reversal potential of −65 mV is very close to the stable fixed point inside the red curve for the unstable limit cycle. A fraction of the population gets trapped inside the unstable limit cycle and skip a cycle. The slowest neurons tend to remain trapped. This explains the greater tendency for suppression in type 2 neurons for versus hyperpolarizing inhibition.

Movie 3. Dynamics of the type 1 population with shunting inhibition. Plots and lines are the same as for Movie 1. 10.1523/ENEURO.0464-19.2020.video.3

Movie 4. Dynamics of the type 2 population with shunting inhibition. Plots and lines are the same as for Movie 1. 10.1523/ENEURO.0464-19.2020.video.4

### Phase amplitude coupling

In the hippocampus, γ power is maximal when nested in theta oscillations (Bragin et al., 1995). In order to determine the relative abilities of networks of neurons with type versus type 2 excitability to produce theta-nested γ, we drove these networks with perfectly sinusoidal inhibitory waveforms at a fixed frequency in the theta range, in the presence of the constant heterogeneous depolarizing bias currents distributed as described in Materials and Methods. The depolarization mimics tonic activation of metabotropic glutamatergic/cholinergic receptors. PV+ basket cells in freely moving rats fire at ∼7 Hz during low oscillatory periods, but that rate triples to 21 Hz during theta oscillations (Lapray et al., 2012), presumably due to greater tonic excitation. The sinusoidal drive mimics phasic inhibition from the septum.

### Phase amplitude coupling, hyperpolarizing inhibition

**Figure 7.**
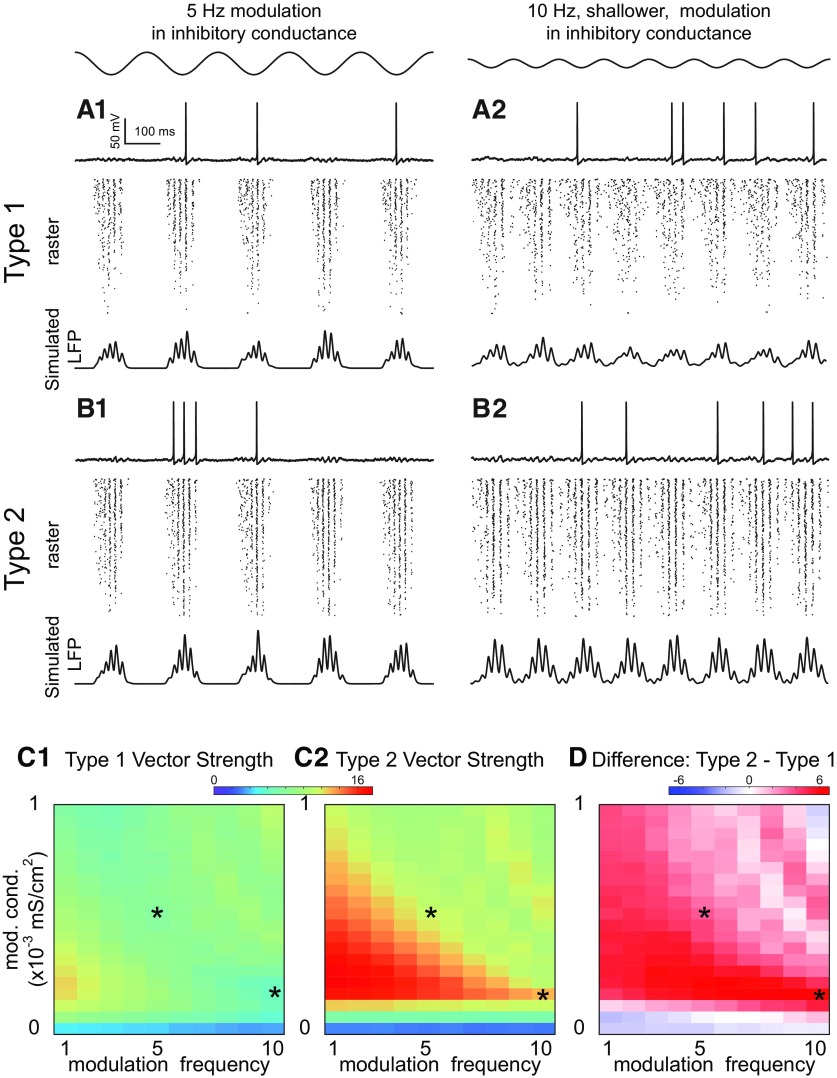
Theta phase γ amplitude modulation of heterogeneous inhibitory interneuronal networks with hyperpolarizing inhibition. Two examples of sinusoidal modulation of these networks, with a 5-Hz sinusoidal modulation in inhibitory conductance shown at top left and a shallower 10-Hz sinusoidal modulation shown at top right (conductance $g_{ij}=0.1\;mS/cm^{2}$, noise SD $\sigma =3\mu A/cm^{2}$). ***A***, Type 1 networks. ***A1***, Five-Hertz modulation. ***A2***, Ten-Hertz modulation. Top, Representative sparsely firing single neuron type 1 traces with subthreshold oscillations due to network activity. Middle, Raster plots for 300 neurons with faster neurons (based on I app) shown at the top. Bottom, Simulated LFP consisting of summated inhibitory currents throughout the network. ***B***, Type 2 networks. ***B1***, Five-Hertz modulation. ***B2***, Ten-Hertz modulation. Top, Representative sparsely firing single neuron type 2 traces with subthreshold oscillations due to network activity. Middle, Raster plots for 300 neurons with faster neurons (based on I app) shown at the top. Bottom, Simulated LFP consisting of summated inhibitory currents throughout the network. ***C***, Heatmaps for 2D parameter space of modulation depth (given in terms of the peak of the sinusoidal conductance waveform) and frequency. Asterisks show parameter values from ***A***, ***B***. ***C1***, Vector strengths for type 1 networks. ***C2***, Vector strengths for type 2 networks. ***C3***, Difference of vector strengths for type 2 and type 1 networks. Hot colors indicate a difference greater than zero. The difference is well above zero in the lower left-hand corner plot meaning that the vector strength for type 2 networks is higher.

Figure 7 gives examples of sinusoidal modulation at theta frequency of nested γ oscillation. The time course of the modulation for both types of networks is the same and is given at the top of the figure. In Figure 7*A1*,*A2*,*B1*,*B2*, each panel has a representative single neuron trace at the top, a raster plot in the middle, and a simulated LFP using the total inhibitory synaptic current summed across the network at the bottom. Both the raster lots and the simulated LFP show more neurons are recruited into the γ rhythm in type 2 (Fig. 7*B*) networks compared with type 1 (Fig. 7*A*), both for a slower deeper modulation on the left, and a shallower faster modulation on the right. The noise and conductance parameters were selected in a regime in which all four types of networks synchronized well. The attributes of the network oscillations for the selected parameter regime are given in Table 3. In additional sets of simulations (data not shown), we confirmed that the results shown below are qualitatively similar for wide range of synaptic conductance and noise levels and are not specific for the chosen values. Figure 7*C* shows the results in the 2D parameter space of modulation depth and modulation frequency, with the color in Figure 7*C1*,*C2* indicating the un-normalized vector strength as described in Materials and Methods. If the vectors were normalized to reflect only how tightly locked the LFP envelope was to the theta drive, the two types perform equally. Removing the normalization reveals the greater recruitment of the population into the nested γ in type 2 networks. The heatmaps in Figure 7*C1*,*C2* show that low frequencies and shallow modulations are most effective, especially for type 2. The heatmap in Figure 7*D* shows that type 2 always outperforms type 1 because the red color is always greater than zero.

**Table 3:** Characteristics of network steady-state oscillations with the parameters used in simulations with theta modulation: conductance $g_{ij}=0.1\;mS/cm^{2}$ **, noise SD** $\sigma =3\mu A/cm^{2}$

	Hyperpolarizing inhibition		Shunting inhibition	
	Type 1	Type 2	Type 1	Type 2
R	0.8	0.88	0.75	0.67
Mean participation	0.2	0.27	0.22	0.17
CV of participation	0.81	0.64	0.64	0.65
Total suppression	0.15	0.03	0.04	0.04

Almost no theta modulation is evident in the membrane potential traces of individual neurons Figure 7*A*,*B*. As the network recovers from inhibitory modulation, the firing rate in the population increases, therefore network inhibition fills in when the external inhibitory drive wanes. This feedback mechanism is also responsible for keeping neurons near the bifurcation at minimal inhibition in steady-state oscillations (Figs. 4*A1*,*B1*, 6*A1*,*B1*).

**Figure 8.**
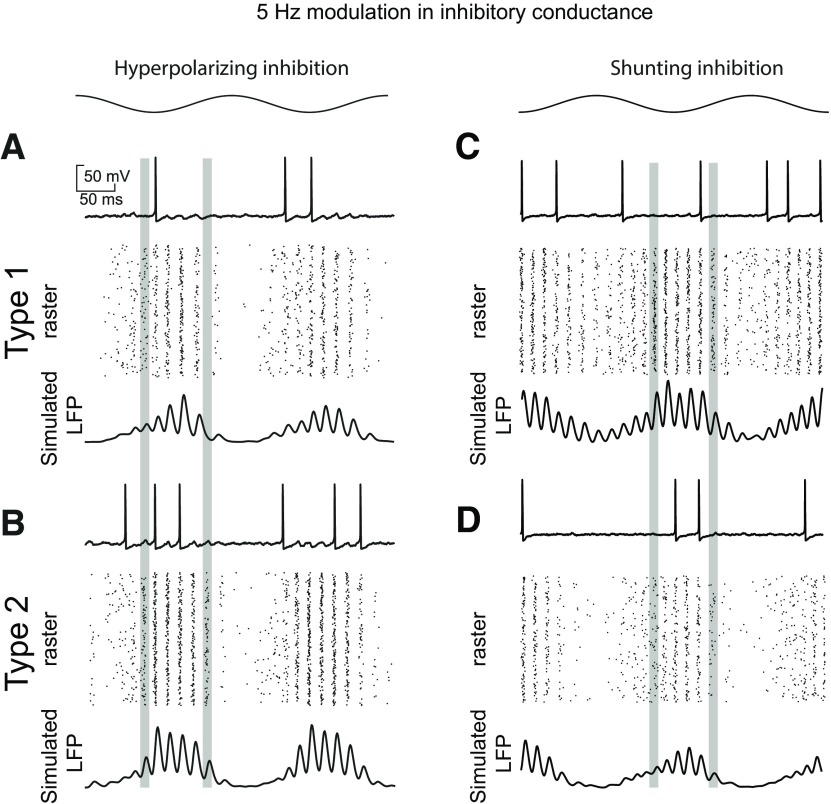
Speed of recruitment of theta-nested γ in homogeneous inhibitory networks with versus shunting inhibition. ***A***, Homogeneous inhibitory network with hyperpolarizing inhibition showing that type 2 neurons are recruited more quickly into theta-nested γ (leftmost vertical gray bar) and persist longer (rightmost gray bar). ***A1***, Type 1. ***A2***, Type 2. ***B***, Homogeneous inhibitory network with hyperpolarizing inhibition showing that type 1 neurons are recruited more quickly into theta-nested γ (leftmost vertical gray bar) and persist longer (rightmost gray bar). ***B1***, Type 1. ***B2***, Type 2.

In order to explain the superior performance of type 2 for hyperpolarizing inhibition, we can refer back to the phase plane in Figure 4*A*. In order to synchronize, type 1 networks rely on the accumulation of inhibition as because a minimum number of neurons must escape through the slow channel to create the stable fixed point that causes some neurons to skip. In contrast, the intrinsic dynamics of a Hopf bifurcation shown in Figure 4*B* create the stable fixed point that causes some neurons to skip. Moreover, there is no slow channel to delay spiking for type 2 neurons, thus spiking can be recruited more quickly. We hypothesize that the improved recruitment of neurons into the γ oscillation in type 2 networks was because type 1 networks need more time to establish synchrony. We tested this idea in a network in which all neurons had the same applied bias current and therefore the same intrinsic frequency (Fig. 8*A*). The speed of synchronization is clearer for the homogeneous frequency case because participation is more uniform. A head-to-head comparison over two cycles of sinusoidal reveals that indeed synchronization begins earlier in the type 2 homogeneous network compared with the type 1 homogeneous network (see leftmost vertical gray bar), which accounts in part for the larger vector strength for theta phase γ amplitude coupling for type 2 with hyperpolarizing inhibition. The second gray bar shows that the synchrony persists longer for type 2 as well.

The superiority of type 2 for hyperpolarizing inhibition derives from the ability to more quickly recruit the activity of sufficient neurons to establish synchrony at γ frequencies. The presumption is that the interneurons are in an excited, oscillatory state during theta activity, and are rhythmically inhibited by the septum. For modulation that is too shallow, γ activity is ongoing and theta power is weak (Fig. 7 *C1*,*C2*, bottom blue strips). For modulation that is sufficiently deep to establish theta power, the modulation must be sufficiently slow or shallow that there are still long enough windows of time above the spiking threshold to recruit enough active neurons to establish γ synchrony (Fig. 7*C1*,*C2*, red areas in bottom left half).

### Phase amplitude coupling, shunting inhibition

The faster recruitment of type 2 oscillators did not persist for homogeneous networks with shunting inhibition (Fig. 8*B*). Instead, type 1 oscillators are recruited more quickly into nested theta γ because shunting inhibition clamps neurons closer to the spiking threshold (Fig. 6*C1* vs Fig. 4*C1*). Type 2 networks clearly lose their superiority, likely because of the tendency of type 2 oscillators with shunting inhibition to remain trapped at the fixed point inside the unstable limit cycle (Fig. 6*B1*,*C2*).

**Figure 9.**
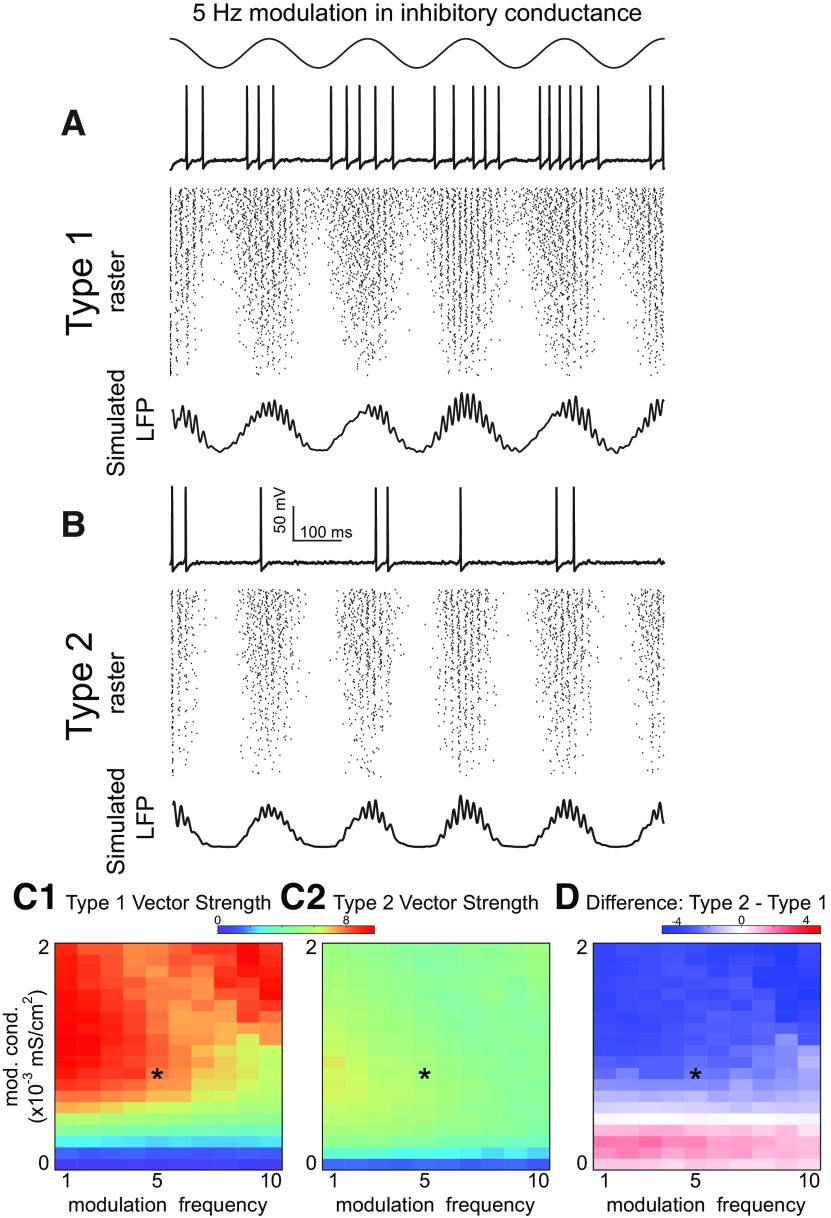
Theta phase γ amplitude modulation of heterogeneous inhibitory interneuronal networks with shunting inhibition. One example of sinusoidal modulation of these networks, with a 5-Hz sinusoidal modulation in inhibitory conductance shown at the top for the same synaptic conductance and noise SD as in Figure 7. ***A***, Type 1 networks modulation. Top, Representative sparsely firing single neuron type 1 traces with subthreshold oscillations due to network activity. Middle, Raster plots for 300 neurons with faster neurons (based on I app) shown at the top. Bottom, Simulated LFP consisting of summated inhibitory currents throughout the network. ***B***, Type 2 networks. Top, Representative sparsely firing single neuron type 2 traces with subthreshold oscillations due to network activity. Middle, Raster plots for 300 neurons with faster neurons (based on I app) shown at the top. Bottom, Simulated LFP consisting of summated inhibitory currents throughout the network. ***C***, Heatmaps for 2D parameter space of modulation depth (given in terms of the peak of the sinusoidal conductance waveform) and frequency. Asterisk shows the parameter values from ***A***, ***B***. ***C1***, Vector strengths for type 1 networks. ***C2***, Vector strengths for type 2 networks. ***C3***, Difference of vector strengths for type versus type 1 networks. Cool colors indicate a difference less than zero. The difference is below zero in this plot, meaning that the vector strength for type 2 networks is higher, except at the bottom where the vector strength is relatively small.

Figure 9*A*,*B* shows examples of type 1 and 2 networks with shunting inhibition. Just as switching from hyperpolarizing to shunting inhibition nullified the advantage of type 2 network in robustness to suppression during steady state synchrony shown in Figures 3, 5, changing the inhibition from hyperpolarizing to shunting nullifies the advantage of type 2 for phase amplitude coupling as predicted by Figure 8. In fact, Figure 9*D* shows that type 1 networks have better phase amplitude coupling for deep modulations (blue indicates a type 2–type 1 difference is less than zero). A minimum amplitude of conductance modulation strength is required to observe superior type 1 PAC; at low modulation amplitudes with small vector strength values (Fig. 9*C1*,*C2*, blue at the bottom) type 2 performs slightly better. The tendency of the unstable limit cycle in Figure 6*B1* to trap trajectories clearly reduces the speed of synchronization of type 2, and is likely responsible for its poorer performance with shunting inhibition.

### Robustness of both mechanisms to different types of heterogeneity and gap junctional connectivity

**Figure 10.**
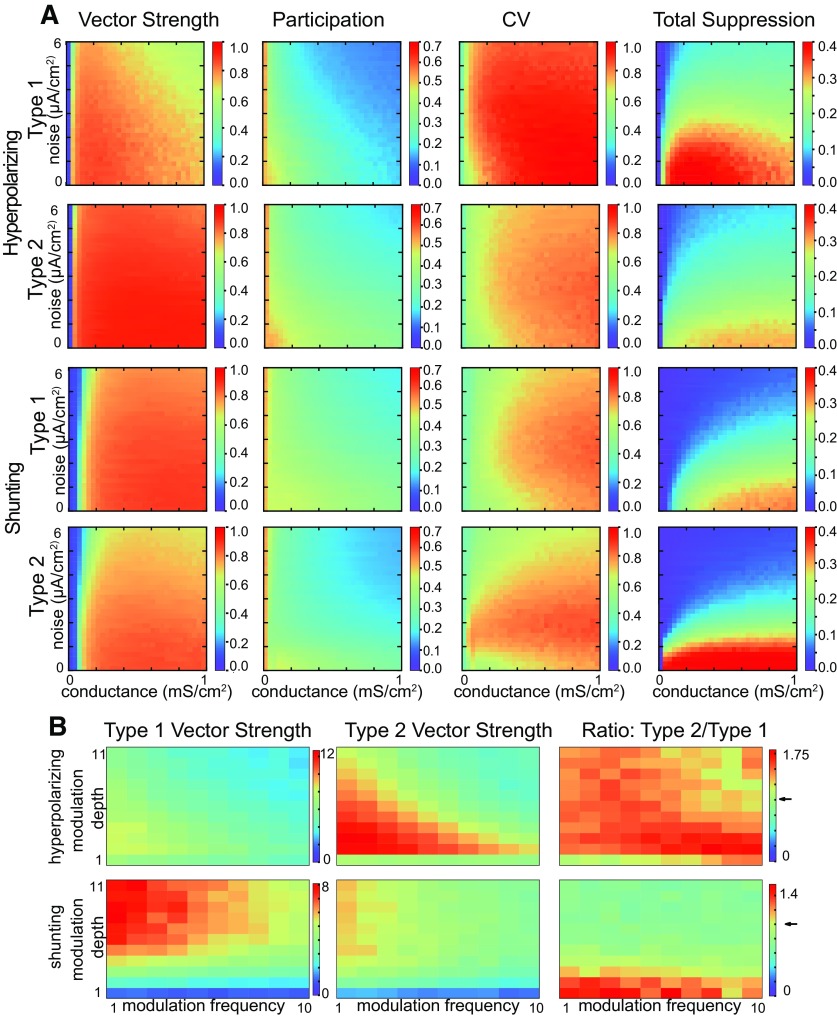
Replication of results with additional heterogeneity in intrinsic dynamics. In addition to a uniform distribution of applied currents $I_{0,i}=[2,\;3.7]\;\mu A/cm^{2}$, heterogeneity in $F_{i}=1.04\pm 0.4$ normally distributed was introduced in the population. $F_{i}$ gives a range of cutoff frequencies from 10 to 65 Hz for type 2. ***A***, Steady state synchronization 2D parameter sweep in conductance strength and noise SD. Heatmaps from left to right, Vector strength *R* of synchronization of individual spikes with the network oscillation, average participation for neurons that are not totally suppressed calculated as the mean µ_Fr/Fnet_ of the frequency of spiking neurons normalized by the population frequency, the CV of the participation CV_Fr/Fnet_, and the fraction of completely suppressed neurons. The top two rows show that the major result from Figure 3 for networks with hyperpolarizing inhibition without the additional heterogeneity is preserved: there is more variability in the CV of participation and more neurons completely suppressed for type versus type 2. Type 2 networks are still able to achieve stronger R>0.9 synchronization almost everywhere, whereas type 1 networks can reach R≈0.85 in a small subregion and show moderate synchronization R∈[0.6, 0.8] in the rest of parameter space. Heterogeneity in the neuron dynamics reduces average participation in network oscillation compared with Figure 3, dramatically increases variability in participation for type 1 networks (CV from 0.8 to 1.0 almost everywhere), and increases total suppression for both types. The next two rows show that the major result from Figure 5 for networks with hyperpolarizing inhibition without the additional heterogeneity is preserved: more neurons completely suppressed for type versus type 1. ***B***, Theta phase γ amplitude coupling. Heatmaps for 2D parameter space of modulation depth and frequency. Cool colors indicate a ratio less than one in the heatmaps for the ratio. The ratio is always above one for hyperpolarizing inhibition meaning that the vector strength for type 2 networks is higher, as in Figure 7. The ratio is below one for shunting inhibition as in Figure 9, meaning that the vector strength for type 2 networks is higher, except at the bottom where the vector strength is small.

In Figures 3, 5, the applied current was varied to simulate heterogeneity in excitatory drive, as is typical (Wang and Buzsáki, 1996; White et al., 1998). However, in addition to having different levels of excitatory input, different interneurons have different F/I curves. For type 1, the slope of the F/I curve is variable, and for type 2 neurons the cutoff frequency is also variable. We incorporated this additional dimension of variability into our simulations using a scale factor for the model dynamics as described in Materials and Methods. In order to determine the effect of heterogeneity in this parameter, we scaled the dynamics of both variables for each neuron to achieve uniformly distributed cutoff frequencies in the range from 10 to 60 Hz for type 2 neurons, and used the same range of scale factors for type 1 neurons. We then redid the heatmaps for each type with hyperpolarizing (Fig. 10*A*, top two rows) and with shunting inhibition (Fig. 10*A*, next two rows). Although both types show a reduction in synchronization, the qualitative results that type 2 networks are more resistant to suppression for hyperpolarizing but not shunting inhibition still hold. Figure 10*B* shows that the PAC tendencies for type versus type 2 are also preserved under the additional heterogeneity used in Figure 10*A*.

We ran additional simulations (data not shown) to determine the effect of electrical combined with chemical synapses since electrical coupling between interneurons has been observed in some brain areas (Fukuda and Kosaka, 2000; Gibson et al., 2005). For sufficiently strong gap junctional conductance and sufficiently high connection probability, all interneurons fire together in one-to-one spike synchrony, which is likely nonphysiological. For intermediate values of conductance and connectivity, the results transition between the suppression of subsets interneurons on certain cycles (as shown in Figs. 3, 5, 7, 9, 10), and complete synchrony with very strong gap junctional connectivity. The contributions of chemical and electrical synapses to interneuronal synchrony are variable between subregions, so the additional synchrony expected from gap junctions should also vary without qualitatively affecting our conclusions.

## Discussion

### Summary of results

We present two major results. The first is that networks of heterogeneous neural oscillators with type 2 excitability are more resistant to suppression of the slower oscillators than those with type 1 excitability when they are coupled with hyperpolarizing inhibition but not with shunting inhibition. The second result is that theta phase to γ amplitude coupling is more strongly recruited in the type 2 networks, again for hyperpolarizing but not shunting inhibition. Moreover, we provide mechanistic explanations for these phenomena, which frame conditions for the generality of these results.

The tendency of slower type 1 neurons to be more suppressed than type 2 neurons for hyperpolarizing inhibition relies on two principles. The first is the creation by inhibition of a slow channel near the SNIC bifurcation for type 1. The channel serves to line the fixed points of the heterogeneous oscillators up along the slow nullcline and along the direction of motion of the fast subsystem. This arrangement clearly favors the faster neurons since their fixed points are to the right and closer to escaping from the slow channel that the slower neurons. This tendency is greatly reduced for versus hyperpolarizing inhibition.

The second principle is the circular motion about the subcritical Hopf bifurcation for type 2 neurons and the existence of a boundary in the phase plane between spiking and skipping neurons allows for broader participation in steady state network oscillation for hyperpolarizing, but not shunting, inhibition. The critical assumption is that the stable fixed point just before the Hopf bifurcation lies near the shunt reversal potential, but well above the hyperpolarizing reversal potential. This assumption seems reasonable based on the definitions of hyperpolarizing and shunting inhibition combined with the location of the Hopf bifurcation at the point that positive feedback from the sodium channel destabilizes the rest potential. Thus, hyperpolarizing inhibition pushes all neurons leftward (in the fast direction) and the circular motion brings them downward and into the fast vector fields that give neurons a much more equal chance to fire than type 1. However, shunting inhibition, rather than freeing trajectories from the unstable limit cycle surround the stable fixed point just before the Hopf bifurcation, traps them within that limit cycle, favoring suppression.

These principles apply to the transient synchronization in theta-nested γ as follows. Note that the steady state synchronization and participation are similar between type 1 and type 2. However, type 2 neurons are more quickly recruited into nested theta γ for hyperpolarizing inhibition because their intrinsic dynamics do not require accumulation of synaptic inhibition to split the populations into spiking and skipping groups. Faster recruitment also allows synchronization, which requires a minimum number of active neurons, to persist longer. The phase plane portrait also accounts for the slower recruitment of type 2 neurons with shunting inhibition into nested theta γ and the shorter persistence that accompanies late recruitment. The mechanism is again the tendency of shunting inhibition to clamp the type 2 trajectories inside the unstable limit cycle, which slows recruitment into theta-nested γ.

### Generality and limitations

An important way in which we checked the generality of the results was to introduce a second type of heterogeneity. We varied the slope of the F/I curve for type 1 neurons and both the cutoff frequency and slope for the type 2 neurons by rescaling the temporal dynamics. The cutoff frequency for FS PV+ basket cells (Martínez et al., 2017) had an estimated SD of 40%. Thus, the 10- to 60-Hz range in cutoff frequencies explored should map onto biologically plausible distributions of cutoff frequencies. Qualitatively, our major results were preserved under these manipulations (Fig. 10).

There are limitations to generalizing the results of this study. As stated in the Introduction, we focused on networks with fast GABA_A_ synapses, which tend to favor the suppression regime over phase dispersion. The results on suppression of subsets interneurons on certain cycles (as shown in Figs. 3, 5, 7, 9, 10) ignore the effects of gap junctions. As stated in the results, modest levels of gap junctions do not qualitatively change the results, but very strong coupling would result in much less suppression. We examined a specific parametric range of additive noise and inhibitory conductance strength in which networks of type 1 and type 2 interneurons perform differently. Stronger noise desynchronizes and stronger inhibition promotes suppression, so outside the parameter regime that we examined, neither type of network synchronizes very well, and there is little difference between their performance. Moreover, we examined networks with a specific distribution of synaptic delays. As described in Materials and Methods, the minimum delay was set to avoid the formation of antiphase clusters, which could complicate the analysis.

Another limitation on the generalization of these results is that there are two ways to achieve Hodgkin’s type 2 excitability. One is the classical Hopf bifurcation described here, and the other involves a saddle node that is not on an invariant limit cycle (Izhikevich, 2007; Gerstner et al., 2014). In the latter case, the limit cycle does not originate at the saddle node, but instead coexists with the stable fixed point, resulting in a bistable region and type 2 excitability. The reported cases of type 2 excitability in FS interneurons all appear to involve a Hopf bifurcation (Sciamanna and Wilson, 2011; Tikidji-Hamburyan et al., 2015), and there are no documented instances of a SN off limit cycle in interneuronal populations. Thus, we have restricted our study of type 2 excitability to those resulting from a Hopf bifurcation. If the region of bistability for a saddle node off limit cycle bifurcation is small, it is not likely to substantially change the dynamics; a full characterization of this alternate route to type 2 excitability is beyond the scope of the current study.

We also ran simulations (data not shown) of mixed networks of type 1 and type 2 interneurons, which tended to behave more like type 1 networks. However, within regions, the excitability type of FS interneurons seems to be consistent. For example, all FS cells in the striatum (Sciamanna and Wilson, 2011) and MEC (Martínez et al., 2017) appear to be type 2, whereas those in CA1 (Wang and Buzsáki, 1996; Ferguson et al., 2013) and dentate gyrus (Vida et al., 2006) seem to be uniformly type 1. Therefore, we compared networks of uniform excitability type within the network. Moreover, we were careful to vary only parameters that did not change the excitability type of the model neurons, because we were interested in isolating the effect of excitability type alone affected synchronization properties.

### Previous comparisons of type 1 versus type 2 excitability

Previously, Rinzel and Ermentrout (1998) contrasted the phase portraits and bifurcation structure for type versus type 2 excitability using the Morris–Lecar (Morris and Lecar, 1981) model in the two regimes. Izhikevich (2007) also showed in the phase plane that various minimal conductance-based models could exhibit saddle node and Hopf bifurcations. Neither study explored the implications of the bifurcation type for synchronization.

In the weak coupling regime, Ermentrout and colleagues (Marella and Ermentrout, 2008; Abouzeid and Ermentrout, 2009) found that type 2 neurons receiving noisy common input synchronize better than type 1. Rich et al. (2017) found that excitatory/inhibitory (E/I) networks with type 2 interneurons were more robust to changes in network connectivity compared with type 1. Synchronization in small networks with type versus type 2 excitability has been contrasted in earlier studies (Achuthan and Canavier, 2009; Wang et al., 2012; Sadeghi and Valizadeh, 2014). None of the type 1 versus type 2 model comparisons cited above were calibrated to match spike shape, shape of the F/I curve, time constant and input resistance across excitability types as were the models in our study. Thus, we extend these previous comparisons so that any difference in network activity is due to the bifurcation type.

Börgers and Walker (2013) compared the synchronization tendencies of either type 1 (Wang and Buzsáki, 1996) or type 2 (Erisir et al., 1999) interneurons embedded in E/I networks with type 1 excitatory neurons. The inhibitory synapses were hyperpolarizing. The modeled E-cells were in the mean-driven regime (with heterogeneous drive) and participated in every γ cycle. In the PING regime, the mean drive for the I cells is also heterogeneous, but below the threshold for repetitive spiking, so they fire when they receive a synchronous volley from the E-cells. Without gap junctions, as the drive to the I-cells was increased to exceed threshold for repetitive firing, the type 1 I-cells desynchronized via phase dispersion of all interneurons, whereas the type 2 neurons desynchronized via suppression of the slower interneurons and phase dispersion among the faster ones. This seems to contradict our results that type 2 neurons are more resistant to suppression in networks with hyperpolarizing inhibition. However, there is no contradiction with our results. The results presented in our study apply when the population is in a parameter regime in which neurons synchronize their firing with the population, whereas their parameter settings biased their interneurons in an asynchronous regime above the threshold for repetitive firing. The desynchronized I-cells suppressed the E cells. For both types, adding sufficiently strong gap junctions enforces ING synchrony among interneurons (as we also found, see Results, Robustness of both mechanisms to different types of heterogeneity and gap junctional connectivity), but nonetheless leads to suppression of the E-cells via a distinct deterministic mechanism. In this mechanism, the phase delays produced by the synchronized firing of the I-cells exceed the oscillatory period of the mean-driven E cells, resulting in complete suppression of the E cells. It has since been shown that ING can be observed without suppression of the E-cells both experimentally (Vinck et al., 2013, 2015a,b; Perrenoud et al., 2016) and in models (Tikidji-Hamburyan et al., 2019, their Fig. 7A).

### Previous studies on suppression

Early work (Wang and Buzsáki, 1996; Chow et al., 1998; White et al., 1998) on robustness of inhibitory interneuronal synchrony focused of type 1 excitability only. These studies did not include conduction delays, and concluded that hyperpolarizing inhibition and non-physiological low levels of heterogeneity in excitatory drive were required for synchrony; adding delays to the system changes these results. Subsequent studies (Vida et al., 2006) used the same type 1 Wang and Buzsáki model added a ring topography but with conduction delays ranging from 0.7 to 10.5 ms; they found that networks with shunting inhibition were more robust to heterogeneity than with hyperpolarizing inhibition. Their raster plots clearly show more suppression for type 1 for hyperpolarizing compared with shunting inhibition, in agreement with our findings. They repeated their simulation with the type 2 model of Erisir et al. (1999), and obtained similar synchronization results, as in our study. However, they did not show raster plots or report on suppression in the type 2 networks. Another study also used a distribution of delays (from 0.7 to 3.5) to prevent the formation of two cluster solutions (Tikidji-Hamburyan et al., 2019) and stabilize global synchrony by moving the operating point away from the destabilizing discontinuity in the phase resetting curve at 0 and 1.

### Chloride reversal potential may be variable

GABA_A_ receptors are chloride channels. The reversal potential can vary across neurons with different internal chloride concentration due to differential chloride ion handling. The reversal potential for chloride in FS basket cells *in vitro* is about −52 mV (Vida et al., 2006) in the dentate gyrus, which implies shunting inhibition in those cells. Previous studies in CA1 and CA3 concluded that inhibition between basket cells was hyperpolarizing (Buhl et al., 1995; Cobb et al., 1997). However, the Cl- concentration can be modulated (Garand et al., 2019), and there can be intracellular gradients in chloride concentration (Blaesse et al., 2009). Therefore, there is sufficient uncertainty regarding the precise reversal potential of chloride at synapses between interneurons to warrant a systematic study of both types of inhibition. Moreover, there may be variability between brain regions; it is conceivable that the reversal potential for mutual inhibition and the excitability type of the interneurons could be co-regulated to optimize synchronization properties.

### Support for cycle skipping in the mean-driven oscillatory regime to enforce synchrony

Pairs of FS interneurons with reciprocal inhibitory (but not electrical) coupling can synchronize *in vitro* with millisecond precision (Hu et al., 2011, their Fig. 4; Gibson et al., 2005, their Fig. 13) when they are both depolarized into an oscillatory regime by current injection. On cycles in which synchrony is not tight, one neuron suppresses the other, resulting in cycle skipping. Cycle skipping characterized by bi- or multi-modal ISI histograms in FS interneurons was observed in pharmacologically induced γ in a slice preparation (Hájos et al., 2004, their Fig. 4B,C) and in computational PING (Economo and White, 2012) and ING models with both type 1 (Buzsáki et al., 2004), and type 2 neurons (Tikidji-Hamburyan et al., 2015). Moreover, there are instances of inhibitory neurons in the oscillatory regime *in vivo*. For example, a subset of FS interneurons were recently identified in mouse barrel cortex in vivo whose firing was clocklike at γ frequency (Shin and Moore, 2019). Of even greater relevance, in mouse striatum, the subset of FS neurons that were strongly locked to transient 50-Hz γ oscillations in vivo exhibited clear bimodal peaks in their ISI histogram (at 20 and 40 ms; Berke, 2009). CA3 FS cells fired at 21 Hz during 45-Hz γ, thus they fired roughly on every other cycle (Tukker et al., 2013), but no histograms were provided.

Cycle skipping provides a mechanism by which coupled oscillator models can produce tightly synchronized firing with sparse firing in individual neurons. This mechanism is distinct from a clustering mechanism that was advanced to introduce robustness of population synchrony. In that mechanism, interneurons did not participate on every γ cycle because synchronized subclusters fired in sequence (Tiesinga and José, 2000). Another study (Tikidji-Hamburyan et al., 2015) found that cycle skipping was more prominent in type versus type 1 networks with hyperpolarizing inhibition, but a fair comparison across the parameter space with matched models was not performed. Cycle skipping allows robustness to heterogeneity in excitatory drive and connectivity for both type 1 and type 2 networks, overcoming a perceived weakness of coupled oscillator models of network γ. Since γ frequency is variable and often modulated by theta, physiological ISI histograms from FS interneurons during episodes of γ are expected to be less sharply peaked than the examples in Figures 3*A*,*B*, 5*A*,*B* for steady, sustained γ oscillations. However, if FS interneurons are in the mean-driven oscillatory regime during γ episodes *in vivo*, a clear neural signature of γ resulting from cycle skipping interactions between interneurons in the mean driven regime would be potentially unimodal ISI histograms in the fastest interneurons and bi or even multimodal histograms in other interneurons.

### Therapeutic directions for PAC and cognition

The hippocampal theta rhythm has been shown to be necessary for spatial learning by rats in a water maze (McNaughton et al., 2006). Phase amplitude coupling between theta and γ likely plays an important role in cognition (Fell and Axmacher, 2011), and we have shown here that type 2 excitability in interneurons strengthens phase amplitude coupling between theta and γ rhythms for hyperpolarizing but weakens it for shunting inhibition. This suggests the possibility that interneurons could modulate their excitability type according to whether inhibition is hyperpolarizing or shunting, or vice versa. Moreover, this suggests a therapeutic strategy to manipulate excitability type, and thereby theta-nested γ phase amplitude coupling, by targeting currents active in FS interneurons in the subthreshold regime to tip the balance toward outward currents.

The balance of inward and outward currents in the voltage range traversed between action potentials during the ISI determines whether a neuron exhibits type 1 or type 2 excitability, and is easily modifiable by altering this balance (Golomb et al., 2007; Prescott et al., 2008). For type 2, outward currents predominate at steady state in this region of subthreshold membrane potential, but are activated more slowly than the inward currents. Therefore, the dwell time in this region of membrane must be brief so that the outward current does not equilibrate and stop the depolarization caused by the inward currents between spikes. There is a maximum ISI for which repetitive spiking can be supported, hence a minimum frequency below which the neuron cannot sustain repetitive firing. In contrast, inward currents predominate at steady state in the subthreshold range on membrane potentials spanned by the ISI, therefore arbitrarily low firing rates can be sustained. Decreasing inward or increasing outward currents that have a steady state component during the ISI favors type 2 excitability, and the opposite manipulations favor type 1. These manipulations, depending on whether inhibition is hyperpolarizing or shunting, could increase theta/γ PAC. Increased theta/γ phase amplitude coupling may in turn improve some aspects of cognition.

10.1523/ENEURO.0464-19.2020.ed1 Extended Data 1 Zip file containing neuron code with python wrapper. Download Extended Data 1, ZIP file.

## Synthesis

Reviewing Editor: Upinder S. Bhalla, National Centre for Biological Sciences, Tata Institute of Fundamental Research; Decisions are customarily a result of the Reviewing Editor and the peer reviewers coming together and discussing their recommendations until a consensus is reached. When revisions are invited, a fact-based synthesis statement explaining their decision and outlining what is needed to prepare a revision will be listed below. The following reviewer(s) agreed to reveal their identity: Aditya Gilra, Alireza Valizadeh.

The key concerns have been addressed in this revision. Some minor fixes have

been suggested by the reviewers, including:

Line 25: “A subset of neurons called fast-spiking basket cells tends to inhibit other neurons from firing.”

This is true for all the inhibitory neurons and not only basket cells.

Line 40: “For example, gamma phase-locking in primate area V4 is largely restric

ted to putative fast-spiking (FS) interneurons’

I assume it means only FS interneurons are phase-locked to LFP gamma oscillation... could it be reworded?

Line 72: please rewrite GABA A with A as a subscript.

Line 243: “A steady-state IV curve that intersects the zero current axis in a single point is characteristic of type 2 excitability, whereas one with multiple intersections is characteristic of type 1”.

Is this correct? Am I misunderstanding something? Please check and clarify.

Line 260: “In a homogeneous network, delays on the short end of this range favor a solution with two subclusters in antiphase, whereas delays at the longer end of the range favor global synchrony of a single cluster”.

Is this true for both excitability types? Doesn’t the PRCs matter?

Line 355: “Thus, the mechanism for cycle skipping has a component that results from the intrinsic dynamics of the circular flow around a Hopf”.

Please clarify what is meant of “around a Hopf”

Line 366: “The sharper slope is generic for type 2 versus type 1 because the steepness allows the n-nullcline to avoid the other branches of the V-nullcline, whereas multiple intersections are required for the SNIC in type 1”.

I am not sure “generic” is true. The threshold at which n_inf(V) goes from zero to one, can also change the type of excitability. Please moderate the sentence.

Line 376: “Since the fixed points of the neurons are ordered with slower on the

left and faster on the right,"

Please reword.

Line 380:” The steeper slope for a Hopf versus a SNIC is generic because it is required to switch the stability of the fixed point by moving between branches of the V-nullcline.”

I doubt again if this is the only parameter that can determine the bifurcation type.

Line 622: In the subsection “Previous comparisons of type 1 versus type 2 excitability", the reviewers suggest that other citations are applicable, such as

Sadeghi & Valizadeh, J Comp Neurosci 36(1), 55-66 (2014).

## Author Response

We thank the reviewers for their constructive criticism and helpful suggestions.

1) Line 25: “A subset of neurons called fast-spiking basket cells tends to inhibit other neurons from firing.”

This is true for all the inhibitory neurons and not only basket cells.

The sentence was reworded for clarity:

“We focus on fast spiking basket cells, a subset of inhibitory interneurons.”

2) Line 40: “For example, gamma phase-locking in primate area V4 is largely restric

ted to putative fast-spiking (FS) interneurons"

I assume it means only FS interneurons are phase-locked to LFP gamma oscillation... could it be reworded?

The sentence was also reworded for clarity:

“For example, during a selective visual attention task, phase-locking of spikes in putative fast-spiking (FS) interneurons in primate area V4 to gamma band oscillations in the local field potential (LFP) was strong both during the pre-stimulus period and during visual stimulation. In contrast, gamma phase-locking of putative pyramidal cells was strong only during visual stimulation that re-quires attention (Vinck et al., 2013).”

3) Line 72: please rewrite GABA A with A as a subscript.

Done.

4) Line 243: “A steady-state IV curve that intersects the zero current axis in a single point is characteristic of type 2 excitability, whereas one with multiple intersections is characteristic of type 1”.

Is this correct? Am I misunderstanding something? Please check and clarify.

This sentence was based on Rinzel and Ermentrout’s influential chapter on “Analysis of Neural Excitability and Oscillations” in the book on Methods in Neuronal Modeling by Koch and Segev, in which they show nullclines rather than IV curves. However, the sentence is not strictly accurate, and has been revised as follows:

“A steady state IV curve that intersects the zero current axis in a single point is often associated with type 2 excitability, whereas one with multiple intersections is often associated with type 1; the same is true for the intersections of the nullclines in the phase-plane portrait (Rinzel and Ermentrout, 1998). However, as stated in the Generality and Limitations section, there are exceptions.”

5) Line 260: “In a homogeneous network, delays on the short end of this range favor a solution with two subclusters in antiphase, whereas delays at the longer end of the range favor global synchrony of a single cluster”.

Is this true for both excitability types? Doesn’t the PRCs matter?

For strong, fast coupling the PRCs in response to an IPSP are similar for both excitability types- the coupling is strong enough to be out of the range in which the infinitesimal PRC is informative. We apologize and clarify as follows:

“In a homogeneous network with strong but fast inhibitory synapses, delays on the short end of this range favor a solution with two subclusters in antiphase, whereas delays at the longer end of the range favor global synchrony of a single cluster (Tikidji-Hamburyan et al., 2019).”

6) Line 355: “Thus, the mechanism for cycle skipping has a component that results from the intrinsic dynamics of the circular flow around a Hopf”.

Please clarify what is meant of “around a Hopf”

"Thus, the mechanism for cycle skipping has a component that results from the intrinsic dynamics of the circular flow around a Hopf bifurcation; the circular flow in the phase plane results from oscillatory dynamics in the time domain due to the emergence of complex eigenvalues in the linearized solution evaluated at the fixed point.”

7) Line 366: “The sharper slope is generic for type 2 versus type 1 because the steepness allows the n-nullcline to avoid the other branches of the V-nullcline, whereas multiple intersections are required for the SNIC in type 1”.

I am not sure “generic” is true. The threshold at which n_inf(V) goes from zero to one, can also change the type of excitability. Please moderate the sentence.

The sentence was moderated as follows.

"The sharper slope allows the n-nullcline to avoid the other branches of the V-nullcline enabling a Hopf bifurcation to underlie type 2 excitability, whereas multiple intersections are required for the SNIC with type 1 excitability.

8) Line 376: “Since the fixed points of the neurons are ordered with slower on the

left and faster on the right,"

Please reword.

These two sentences were reworded for clarity:

"The fixed points of the neurons are ordered with those of the neurons with slower firing frequencies (lower bias current) on the left indicating more hyperpolarized fixed points and those corresponding to faster firing frequencies on the right, and the movement of the fixed points as inhibition waxes and wanes does not substantially alter their distribution. Therefore, neurons with the slower intrinsic firing frequencies have a strong tendency to be suppressed with hyperpolarizing inhibition.”

9) Line 380:” The steeper slope for a Hopf versus a SNIC is generic because it is required to switch the stability of the fixed point by moving between branches of the V-nullcline.”

I doubt again if this is the only parameter that can determine the bifurcation type.

The sentence was removed, it is redundant with the sentence revised in point 7 above.

10) Line 622: In the subsection “Previous comparisons of type 1 versus type 2 excitability", other citations should be included, such as

Sadeghi & Valizadeh, J Comp Neurosci 36(1), 55-66 (2014).

We added the following sentence:

"Synchronization in small networks with type 1 versus type 2 excitability has been contrasted in earlier studies (Achuthan and Canavier, 2009; Wang et al., 2012; Sadeghi and Valizadeh, 2014)
