# Supplementary material for: Shunting Inhibition Improves Synchronization in Heterogeneous Inhibitory Interneuronal Networks with Type 1 Excitability Whereas Hyperpolarizing Inhibition Is Better for Type 2 Excitability
Source: eNeuro. 2020 May 8;7(3):ENEURO.0464-19.2020. doi: 10.1523/ENEURO.0464-19.2020 (PMC7210489; doi:10.1523/ENEURO.0464-19.2020)
Supplement: Extended Data 1 — Zip file containing neuron code with python wrapper. Download Extended Data 1, ZIP file. [file enu-eN-TNC-0464-19-s06.zip › CycleSkipping-Type21-blinded/readme.html]

## This NEURON + Python script is associated with a paper:

*XXXX*

**Mechanisms of Inhibitory Interneuronal Network Synchrony for Type 1 versus Type 2 Excitability**

*XXXX*

## Requirements

To use this scripts you need python’s libraries:

- numpy
- scipy
- matplotlib and LaTeX for correct graphical interface

Under Ubuntu or any other Debian based Linux, run *sudo apt-get install python-numpy python-scipy python-matplotlib texlive-full*.

You can use *yum* or *zymm* under RadHad or SUSE based Linux distributions.

## Examples from the paper

To run simulations:

- nrnivmodl
- nrngui -nogui -python network.py **[parameters]**

**[paramters]** for different figures are given below

### Figure 3A

`nrngui -nogui -python network.py /gui=ON /git=False /preview=ON /tv=0,1000 /ncon=\\\'b\\\',0.133 /neuron/Type=1 /neuron/Istdev=1.5e-2 /neuron/Vinit=-50.,20 /neuron/Iapp=\\\'u\\\',0.02,0.037 /synapse/weight=5e-4 /synapse/delay=0.7,3.5 /delay-dist=UNIFORM /synapse/Esyn=-75.0 /ttFFT=False /tracetail=p2eLFP /N2NHI=False /nrnFRhist/range=-0.05,0.85 /nrnFRhist/bins=9 /nrnFRhist/xnorm=True /nrnFRhist/ymax=0.5 /sortbysk=FR /nrnISI=100 /nrnFRhist/part=True`

### Figure 3B

`nrngui -nogui -python network.py /gui=ON /git=False /preview=ON /tv=0,1000 /ncon=\\\'b\\\',0.133 /neuron/Type=2 /neuron/Istdev=1.5e-2 /neuron/Vinit=-50.,20. /neuron/Iapp=\\\'u\\\',0.02,0.037 /synapse/weight=5e-4 /synapse/delay=0.7,3.5 /delay-dist=UNIFORM /synapse/Esyn=-75.0 /ttFFT=False /tracetail=p2eLFP /N2NHI=False /nrnFRhist/range=-0.05,0.85 /nrnFRhist/bins=9 /nrnFRhist/xnorm=True /nrnFRhist/ymax=0.5 /sortbysk=FR /nrnISI=100 /nrnFRhist/part=True`

### Figure 3C 2

`nrngui -nogui -python network.py /git=False /gui=False /preview=False /ncell=300 /ncon=\\\'b\\\',0.133 /neuron/Type=1 /neuron/Vinit=-51.0,20.0 /neuron/Istdev=YYYY\*1e-2 /neuron/Iapp=\\\'u\\\',0.02,0.037 /synapse/weight=XXX\*1e-2 /synapse/delay=0.7,3.5 /delay-dist=UNIFORM /synapse/Esyn=-75.0 /tstop=2500 /cliptrn=500` where XXX is a synaptic conductance and YYYY is a level of noise. The scale 1e-2 converts nA into uA/cm2 and uS into mS/cm2.

### Figure 3D 2

`nrngui -nogui -python network.py /git=False /gui=False /preview=False /ncell=300 /ncon=\\\'b\\\',0.133 /neuron/Type=2 /neuron/Vinit=-51.0,20.0 /neuron/Istdev=YYYY\*1e-2 /neuron/Iapp=\\\'u\\\',0.02,0.037 /synapse/weight=XXX\*1e-2 /synapse/delay=0.7,3.5 /delay-dist=UNIFORM /synapse/Esyn=-75.0 /tstop=2500 /cliptrn=500` where XXX is a synaptic conductance and YYYY is a level of noise.

### Figure 4A and supplementary movie sp1-20190619163238.mp4 1

`nrngui -nogui -python network.py /gui=ON /git=False /preview=ON /sortbysk=I /tv=400,500 /ncell=300 /ncon=\\\'b\\\',0.133 /neuron/Type=1 /neuron/Istdev=1.5e-2 /neuron/Vinit=-68 /neuron/Iapp=\\\'u\\\',0.02,0.037 /synapse/weight=5e-4 /synapse/delay=0.7,3.5 /delay-dist="UNIFORM" /ttFFT=False /tracetail=p2eLFP /N2NHI=False /pop-pp-view=True /PhaseLims=(-76,-20),(0.3,0.6) /pop-pp-view-color=True`

### Figure 4B and supplementary movie sp2-20190619165631.mp4 1

`nrngui -nogui -python network.py /gui=ON /git=False /preview=ON /sortbysk=I /tv=400,500 /ncell=300 /ncon=\\\'b\\\',0.133 /neuron/Type=2 /neuron/Istdev=1.5e-2 /neuron/Vinit=-68 /neuron/Iapp='u',0.02,0.037 /synapse/weight=5e-4 /synapse/delay=0.7,3.5 /delay-dist="UNIFORM" /ttFFT=False /tracetail=p2eLFP /N2NHI=False /pop-pp-view=True /PhaseLims=(-76,-20),(0.3,0.6) /pop-pp-view-color=True`

### Figure 5A

`nrngui -nogui -python network.py /gui=ON /git=False /preview=ON /tv=0,1000 /ncon=\\\'b\\\',0.133 /neuron/Type=1 /neuron/Istdev=1.5e-2 /neuron/Vinit=-50.,20 /neuron/Iapp=\\\'u\\\',0.02,0.037 /synapse/weight=15e-4 /synapse/delay=0.7,3.5 /delay-dist=UNIFORM /synapse/Esyn=-65.0 /ttFFT=False /tracetail=p2eLFP /N2NHI=False /nrnFRhist/range=-0.05,0.85 /nrnFRhist/bins=9 /nrnFRhist/xnorm=True /nrnFRhist/ymax=0.5 /sortbysk=FR /nrnISI=100 /nrnFRhist/part=True`

### Figure 5B

`nrngui -nogui -python network.py /gui=ON /git=False /preview=ON /tv=0,1000 /ncon=\\\'b\\\',0.133 /neuron/Type=2 /neuron/Istdev=1.5e-2 /neuron/Vinit=-50.,20. /neuron/Iapp=\\\'u\\\',0.02,0.037 /synapse/weight=15e-4 /synapse/delay=0.7,3.5 /delay-dist=UNIFORM /synapse/Esyn=-65.0 /ttFFT=False /tracetail=p2eLFP /N2NHI=False /nrnFRhist/range=-0.05,0.85 /nrnFRhist/bins=9 /nrnFRhist/xnorm=True /nrnFRhist/ymax=0.5 /sortbysk=FR /nrnISI=100 /nrnFRhist/part=True`

### Figure 5C 2

`nrngui -nogui -python network.py /git=False /gui=False /preview=False /ncell=300 /ncon=\\\'b\\\',0.133 /neuron/Type=1 /neuron/Vinit=-51.0,20.0 /neuron/Istdev=YYYY\*1e-2 /neuron/Iapp=\\\'u\\\',0.02,0.037 /synapse/weight=XXX\*1e-2 /synapse/delay=0.7,3.5 /delay-dist=UNIFORM /synapse/Esyn=-65.0 /tstop=2500 /cliptrn=500` where XXX is a synaptic conductance and YYYY is a level of noise.

### Figure 5D 2

`nrngui -nogui -python network.py /git=False /gui=False /preview=False /ncell=300 /ncon=\\\'b\\\',0.133 /neuron/Type=2 /neuron/Vinit=-51.0,20.0 /neuron/Istdev=YYYY\*1e-2 /neuron/Iapp=\\\'u\\\',0.02,0.037 /synapse/weight=XXX\*1e-2 /synapse/delay=0.7,3.5 /delay-dist=UNIFORM /synapse/Esyn=-65.0 /tstop=2500 /cliptrn=500`

where XXX is a synaptic conductance and YYYY is a level of noise.

### Figure 6A and supplementary movie sp3-20190619103132.mp4 1

`nrngui -nogui -python network.py /gui=ON /git=False /preview=ON /sortbysk=I /tv=400,500 /ncell=300 /ncon=\\\'b\\\',0.133 /neuron/Type=1 /neuron/Istdev=1.5e-2 /neuron/Vinit=-68 /neuron/Iapp=\\\'u\\\',0.02,0.037 /synapse/weight=15e-4 /synapse/delay=0.7,3.5 /delay-dist=UNIFORM /synapse/Esyn=-65. /ttFFT=False /tracetail=p2eLFP /N2NHI=False /pop-pp-view=True /PhaseLims=(-76,-20),(0.3,0.6) /pop-pp-view-color=True`

### Figure 6B and supplementary movie sp4-20190619104959.mp4 1

`nrngui -nogui -python network.py /gui=ON /git=False /preview=ON /sortbysk=I /tv=400,500 /ncell=300 /ncon=\\\'b\\\',0.133 /neuron/Type=2 /neuron/Istdev=1.5e-2 /neuron/Vinit=-68 /neuron/Iapp=\\\'u\\\',0.02,0.037 /synapse/weight=15e-4 /synapse/delay=0.7,3.5 /delay-dist=UNIFORM /synapse/Esyn=-65. /ttFFT=False /tracetail=p2eLFP /N2NHI=False /pop-pp-view=True /PhaseLims=(-76,-20),(0.3,0.6) /pop-pp-view-color=True`

### Figure 7 A1

`nrngui -nogui -python network.py /neuron/Type=1 /neuron/Vinit=-51.0,20.0 /neuron/Istdev=0.03 /neuron/Iapp=\\\'u\\\',0.02,0.037 /git=False /gui=ON /preview=ON /ncell=300 /ncon=\\\'b\\\',0.133 /synapse/weight=0.001 /synapse/Esyn=-75.0 /synapse/delay=0.7,3.5 /delay-dist=UNIFORM /tracetail=p2eLFP /p2eLFP_max=250 /ttFFT=False /PAC-VS=True /sortbysk=I /singmod/E=-75.0 /singmod/tstart=0 /singmod/tstop=2000.0 /tv=0.,2000.0 /singmod/gmax=5.e-06 /singmod/per=200.0` ### Figure 7 A2 `nrngui -nogui -python network.py /neuron/Type=2 /neuron/Vinit=-51.0,20.0 /neuron/Istdev=0.03 /neuron/Iapp=\\\'u\\\',0.02,0.037 /git=False /gui=ON /preview=ON /ncell=300 /ncon=\\\'b\\\',0.133 /synapse/weight=0.001 /synapse/Esyn=-75.0 /synapse/delay=0.7,3.5 /delay-dist=UNIFORM /tracetail=p2eLFP /p2eLFP_max=250 /ttFFT=False /PAC-VS=True /sortbysk=I /singmod/E=-75.0 /singmod/tstart=0 /singmod/tstop=2000.0 /tv=0.,2000.0 /singmod/gmax=5.e-06 /singmod/per=200.0`

### Figure 7 B1

`nrngui -nogui -python network.py /neuron/Type=1 /neuron/Vinit=-51.0,20.0 /neuron/Istdev=0.03 /neuron/Iapp=\\\'u\\\',0.02,0.037 /git=False /gui=ON /preview=ON /ncell=300 /ncon=\\\'b\\\',0.133 /synapse/weight=0.001 /synapse/Esyn=-75.0 /synapse/delay=0.7,3.5 /delay-dist=UNIFORM /tracetail=p2eLFP /p2eLFP_max=250 /ttFFT=False /PAC-VS=True /sortbysk=I /singmod/E=-75.0 /singmod/tstart=0 /singmod/tstop=2000.0 /tv=0.,2000.0 /singmod/gmax=2.e-06 /singmod/per=100.0`

### Figure 7 B2

`nrngui -nogui -python network.py /neuron/Type=2 /neuron/Vinit=-51.0,20.0 /neuron/Istdev=0.03 /neuron/Iapp=\\\'u\\\',0.02,0.037 /git=False /gui=ON /preview=ON /ncell=300 /ncon=\\\'b\\\',0.133 /corefunc=24 /synapse/weight=0.001 /synapse/Esyn=-75.0 /synapse/delay=0.7,3.5 /delay-dist=UNIFORM /tracetail=p2eLFP /p2eLFP_max=250 /ttFFT=False /PAC-VS=True /sortbysk=I /singmod/E=-75.0 /singmod/tstart=0 /singmod/tstop=2000.0 /tv=0.,2000.0 /singmod/gmax=2.e-06 /singmod/per=100.0`

### Any point on heatmap Figure 7 C1 2

`nrngui -nogui -python network.py /neuron/Type=1 /neuron/Vinit=-51.0,20.0 /neuron/Istdev=0.03 /neuron/Iapp=\\\'u\\\',0.02,0.037 /git=False /gui=False /preview=False /ncell=300 /ncon=\\\'b\\\',0.133 /synapse/weight=0.001 /synapse/Esyn=-75.0 /synapse/delay=0.7,3.5 /delay-dist=UNIFORM /tracetail=p2eLFP /p2eLFP_max=250 /ttFFT=False /PAC-VS=True /singmod/E=-75.0 /singmod/tstart=0 /singmod/tstop=20000.0 /tstop=20000.0 /singmod/gmax=XXXX\*1e-2 /singmod/per=1000./YYYY`

where XXXX is modulation conductance in *μ**A*/*c**m*2 and YYYY is modulation frequency in Hz

### Any point on heatmap Figure 7 C2 2

`nrngui -nogui -python network.py /neuron/Type=2 /neuron/Vinit=-51.0,20.0 /neuron/Istdev=0.03 /neuron/Iapp=\\\'u\\\',0.02,0.037 /git=False /gui=False /preview=False /ncell=300 /ncon=\\\'b\\\',0.133 /synapse/weight=0.001 /synapse/Esyn=-75.0 /synapse/delay=0.7,3.5 /delay-dist=UNIFORM /tracetail=p2eLFP /p2eLFP_max=250 /ttFFT=False /PAC-VS=True /singmod/E=-75.0 /singmod/tstart=0 /singmod/tstop=20000.0 /tstop=20000.0 /singmod/gmax=XXXX\*1e-2 /singmod/per=1000./YYYY`

where XXXX is modulation conductance in *μ**A*/*c**m*2 and YYYY is modulation frequency in Hz

### Figure 9 A

`nrngui -nogui -python network.py /neuron/Type=1 /neuron/Vinit=-51.0,20.0 /neuron/Istdev=0.03 /neuron/Iapp=\\\'u\\\',0.02,0.037 /git=False /gui=ON /preview=ON /ncell=300 /ncon=\\\'b\\\',0.133 /synapse/weight=0.001 /synapse/Esyn=-65.0 /synapse/delay=0.7,3.5 /delay-dist=UNIFORM /tracetail=p2eLFP /p2eLFP_max=250 /ttFFT=False /PAC-VS=True /sortbysk=I /singmod/E=-65.0 /singmod/tstart=0 /singmod/tstop=2000.0 /tv=0.,2000.0 /singmod/gmax=8.e-06 /singmod/per=200.0`

### Figure 9 B

`nrngui -nogui -python network.py /neuron/Type=2 /neuron/Vinit=-51.0,20.0 /neuron/Istdev=0.03 /neuron/Iapp=\\\'u\\\',0.02,0.037 /git=False /gui=ON /preview=ON /ncell=300 /ncon=\\\'b\\\',0.133 /synapse/weight=0.001 /synapse/Esyn=-65.0 /synapse/delay=0.7,3.5 /delay-dist=UNIFORM /tracetail=p2eLFP /p2eLFP_max=250 /ttFFT=False /PAC-VS=True /sortbysk=I /singmod/E=-65.0 /singmod/tstart=0 /singmod/tstop=2000.0 /tv=0.,2000.0 /singmod/gmax=8.e-06 /singmod/per=200.0`

### Any point on heatmap Figure 9 C1 2

`nrngui -nogui -python network.py /neuron/Type=1 /neuron/Vinit=-51.0,20.0 /neuron/Istdev=0.03 /neuron/Iapp=\\\'u\\\',0.02,0.037 /git=False /gui=False /preview=False /ncell=300 /ncon=\\\'b\\\',0.133 /synapse/weight=0.001 /synapse/Esyn=-65.0 /synapse/delay=0.7,3.5 /delay-dist=UNIFORM /tracetail=p2eLFP /p2eLFP_max=250 /ttFFT=False /PAC-VS=True /singmod/E=-65.0 /singmod/tstart=0 /singmod/tstop=2000.0 /tstop=2000.0 /singmod/gmax=XXXX\*1.e-02 /singmod/per=1000./YYYY`

where XXXX is modulation conductance in *μ**A*/*c**m*2 and YYYY is modulation frequency in Hz

### Any point on heatmap Figure 9 C2 2

`nrngui -nogui -python network.py /neuron/Type=2 /neuron/Vinit=-51.0,20.0 /neuron/Istdev=0.03 /neuron/Iapp=\\\'u\\\',0.02,0.037 /git=False /gui=False /preview=False /ncell=300 /ncon=\\\'b\\\',0.133 /synapse/weight=0.001 /synapse/Esyn=-65.0 /synapse/delay=0.7,3.5 /delay-dist=UNIFORM /tracetail=p2eLFP /p2eLFP_max=250 /ttFFT=False /PAC-VS=True /singmod/E=-65.0 /singmod/tstart=0 /singmod/tstop=2000.0 /tstop=2000.0 /singmod/gmax=XXXX\*1.e-02 /singmod/per=1000./YYYY`

where XXXX is modulation conductance in *μ**A*/*c**m*2 and YYYY is modulation frequency in Hz

### Notes

1. For Figures 4A, 4B, 6A, and 6B, if you click on phase-plot window, you can explore evolution of population dynamics using page-up/page-down keys.
2. Simulations for Figures 3C, 3D, 5C, 5D, 7C, and 9C will not show anything on the screen. All results are saved in the `network.simdb` file. `simdb` has a very simple format: each simulation is a line. Column `:` separates recorded fields. Each field is a couple `key=value` with the equal symbol as a separator. *An example of fields in a simulation record* shows R2-index of network synchronization, spike-per-cycle, and neurons firing rate to network frequency ratio: `/R2-results/R2=0.80846372802:/R2-results/spc=88.1:/R2-results/stdr_Fr/Fnet=0.264655449759`

## Files in this directory

| File | Description |
| --- | --- |
| network.py | main script |
| norm\_translation.py | subroutine for synapses amplitude normalization (wasn’t used in the paper) |
| type21v02.mod | NEURON module for membrane currents of a single neuron |
| innp.mod | noise current generator, writen by Ted Carnevale |
| sinGstim.mod | module for sinusoidal conductance modulation |
| sinIstim.mod | module for sinusoidal current modulation (wasn’t used in the paper) |
